# Sustainability and scalability of a volunteer-based primary care intervention (Health TAPESTRY): a mixed-methods analysis

**DOI:** 10.1186/s12913-017-2468-9

**Published:** 2017-08-01

**Authors:** Monika Kastner, Radha Sayal, Doug Oliver, Sharon E. Straus, Lisa Dolovich

**Affiliations:** 1 Li Ka Shing Knowledge Institute of St. Michael’s Hospital, Toronto, ON Canada; 2 Department of Family Medicine, McMaster University, Hamilton, ON Canada; 3 Faculty of Medicine, University of Toronto, Toronto, ON Canada

**Keywords:** Sustainability, Scalability, Primary care, Survey, Qualitative study

## Abstract

**Background:**

Chronic diseases are a significant public health concern, particularly in older adults. To address the delivery of health care services to optimally meet the needs of older adults with multiple chronic diseases, Health TAPESTRY (Teams Advancing Patient Experience: Strengthening Quality) uses a novel approach that involves patient home visits by trained volunteers to collect and transmit relevant health information using e-health technology to inform appropriate care from an inter-professional healthcare team. Health TAPESTRY was implemented, pilot tested, and evaluated in a randomized controlled trial (analysis underway). Knowledge translation (KT) interventions such as Health TAPESTRY should involve an investigation of their sustainability and scalability determinants to inform further implementation. However, this is seldom considered in research or considered early enough, so the objectives of this study were to assess the sustainability and scalability potential of Health TAPESTRY from the perspective of the team who developed and pilot-tested it.

**Methods:**

Our objectives were addressed using a sequential mixed-methods approach involving the administration of a validated, sustainability survey developed by the National Health Service (NHS) to all members of the Health TAPESTRY team who were actively involved in the development, implementation and pilot evaluation of the intervention (Phase 1: *n* = 38). Mean sustainability scores were calculated to identify the best potential for improvement across sustainability factors. Phase 2 was a qualitative study of interviews with purposively selected Health TAPESTRY team members to gain a more in-depth understanding of the factors that influence the sustainability and scalability Health TAPESTRY. Two independent reviewers coded transcribed interviews and completed a multi-step thematic analysis. Outcomes were participant perceptions of the determinants influencing the sustainability and scalability of Health TAPESTRY.

**Results:**

Twenty Health TAPESTRY team members (53% response rate) completed the NHS sustainability survey. The overall mean sustainability score was 64.6 (range 22.8–96.8). Important opportunities for improving sustainability were better staff involvement and training, clinical leadership engagement, and infrastructure for sustainability. Interviews with 25 participants (response rate 60%) showed that factors influencing the sustainability and scalability of Health TAPESTRY emerged across two dimensions: I) Health TAPESTRY operations (development and implementation activities undertaken by the central team); and II) the Health TAPESTRY intervention (factors specific to the intervention and its elements). Resource capacity appears to be an important factor to consider for Health TAPESTRY operations as it was identified across both sustainability and scalability factors; and perceived lack of interprofessional team and volunteer resource capacity and the need for stakeholder buy-in are important considerations for the Health TAPESTRY intervention. We used these findings to create actionable recommendations to initiate dialogue among Health TAPESTRY team members to improve the intervention.

**Conclusions:**

Our study identified sustainability and scalability determinants of the Health TAPESTRY intervention that can be used to optimize its potential for impact. Next steps will involve using findings to inform a guide to facilitate sustainability and scalability of Health TAPESTRY in other jurisdictions considering its adoption. Our findings build on the limited current knowledge of sustainability, and advances KT science related to the sustainability and scalability of KT interventions.

**Electronic supplementary material:**

The online version of this article (doi:10.1186/s12913-017-2468-9) contains supplementary material, which is available to authorized users.

## Background

Chronic diseases are a significant public health concern [1–3], and were reported by the World Health Organization as the leading cause of death worldwide [3]. Multiple chronic conditions are more prevalent among adults aged 65 years and older, who are not only the fastest growing proportion of our population, but also amongst the highest users of the health care system [4, 5]. Aging is an expensive process, as 10% of seniors who have the most complex health needs account for 60% of the total annual health care spending in many provinces in Canada [6]. By 2030, the increasing number of seniors is projected to cost the Canadian healthcare system $24 billion more annually (50% more than today) [6]. Given these projections, we need to address the delivery of health care services to optimally meet the needs of this population.

Health TAPESTRY (Teams Advancing Patient Experience: Strengthening Quality) was created in response to these challenges and to optimize health care delivery [7]. It uses a novel approach that integrates the involvement of trained volunteers who visit individuals (clients) in their homes and the use e-health technologies via touch screen tablets to identify clients’ health goals and chronic disease risks. Studies suggest that the use of volunteers in health interventions have potential for impact [8–13], particularly because this strategy can respond to the need for low cost, sustainable interventions to address the increasing burden of chronic disease, to promote community engagement, and to address social isolation of older adults. In Health TAPESTRY, data gathered by volunteers during the client visits are transmitted to inform appropriate care from an inter-professional healthcare team consisting of physicians, nurses, system navigators and other health care professionals. As such, it is considered a KT intervention (i.e., it facilitates the uptake of knowledge). Health TAPESTRY delivers the overall intervention with an innovative technology-based system integrated with the McMaster family practice electronic KindredPHR© Personal Health Record (PHR) system. Trained volunteer pairs collect health information over several home visits. The health care team uses the report to design an action plan to address identified client goals and health risks. For example, this may involve a wide range of activities by different health care providers such as optimized diabetes management, medication review, promoting clients’ access to health and community-based programs and services with the help of healthcare navigators, and engaging the volunteers in follow up activities or encouraging clients to use the PHR for chronic disease management or communication with the clinic.

The Health TAPESTRY intervention was conceptualized and developed using an integrated knowledge translation (KT) approach [14]. The approach included a process whereby a wide scope of relevant knowledge users including patients, volunteers, health care providers, leaders of community organizations and experts and stakeholders in geriatric medicine, nursing, allied health, volunteer training, knowledge translation, health services research methods, and information technology worked as a team to create and implement Health TAPESTRY. More specifically its development considered: (1) inclusion of components supported by literature on evidence of effectiveness; (2) theoretical models such as the *Chronic Care Model*, *Behaviour Change*, *Diffusion of Innovation*; (3) developmental evaluation; (4) participatory co-development; (5) formal investigation of sustainability and scalability; (6) iterative pilot testing including evaluation of implementation of the intervention; (7) a pragmatic randomized controlled trial to evaluate the effectiveness and cost effectiveness in older adults; and (8) implementation of an adapted approach in other sites/contexts [7]. Health TAPESTRY was implemented first in an interprofessional primary care practice in Hamilton, Ontario, Canada and pilot-tested using an explanatory mixed methods design to explore its feasibility (e.g., the potential of pairing volunteers, client visits, automation of data transfer from tablets to electronic medical records) [15]; this study involved 11 volunteers, and 11 patients of four family physicians. Findings were used to modify the intervention after which it was evaluated in a large randomized controlled trial (RCT) carried out between 2014 and 2016 with 360 older adults (analysis underway) [16]. We also conducted other smaller feasibility studies to test the potential of Health TAPESTRY in more targeted populations (i.e., people with diabetes and hypertension, high health system users), and utilizing different groups of volunteers (i.e., nursing and medical students who participated as part of a formal for-credit university-level course).

Sustainability is an important implementation outcome and a necessary consideration for scaling up KT interventions such as Health TAPESTRY. In fact, it would be inappropriate to scale-up an intervention that is not sustainable. Identifying the determinants of sustainability is therefore needed to facilitate appropriate scale-up of interventions to ensure that the knowledge that is generated by them can inform optimized decision making for their target end users, and to achieve patient well being and positive health outcomes. Scalability or large-scale adoption is defined as “deliberate effors to increase the impact of health service innovations successfully tested in pilot or experimental projects so as to benefit more people” [17]. It is the vertical diffusion or deliberate, systematic approach to increasing the coverage, range and sustainability of services [18]. Sustainability is commonly defined as: “the degree to which an innovation continues to be used after initial efforts to secure adoption is completed” [19] or becomes a routine part of care delivery and continues to deliver outcomes [20–22]. It has also been defined as an intervention that is in place for more than 1 year after implementation or after the research or project funding period is complete [23]. Sustainability is an important implementation outcome, yet it is seldom considered in research plans or considered early enough in the development process of interventions to be able to address its potential challenges and barriers [24, 25]. Additionally, little research has been done to investigate long-term sustainability of KT interventions [26–29]. Intervention design is often focused on short-term outputs and rarely addresses long-term outcomes in practice and policy domains [24, 25]. This can lead to worse patient outcomes and quality of care [30–32], implementation failure and wasted resources [33–36].

The overall objectives of the current study were to assess the sustainability and scalability potential of the Health TAPESTRY intervention near the beginning of intervention development (prior to the planned RCT) from the perspective of the team who were involved in its development and pilot testing. Specific objectives were to: identify the specific determinants that influence the implementation, sustainability and scale-up of Health TAPESTRY across Canada, and to create a guidance document (addressing barriers) to enable the team to optimize its potential for impact.

## Methods

Our objectives were addressed using a sequential mixed-methods approach conducted in two phases. First, we administered a validated sustainability questionnaire developed by the National Health Service (NHS) Institute (PHASE 1) [37] followed by a qualitative study of one-on-one telephone interviews (PHASE 2). Both phases involved recruiting members of the Health TAPESTRY team (i.e., affiliated researchers, program staff, volunteer organizations) and clinic providers and staff of the McMaster Family Health Team (FHT) in Hamilton, Ontario; volunteers were involved in PHASE 2 only. Research and ethics approval was obtained from the Hamilton Integrated Research Ethics Board as part of the Health TAPESTRY program development and evaluation at McMaster University in Hamilton, Ontario, Canada.

### PHASE 1 – Administration of the NHS sustainability survey

#### Survey instrument

The NHS sustainability model and survey was designed to help teams become aware of potential challenges to the sustainability of a new practice change (such as from an implemented intervention). In the context of their environment, teams affected by the change can determine (through self-assessment using the NHS survey), whether the new practice or change is likely to be sustained, and to prompt timely action to facilitate sustainability [22, 37]. The NHS model and survey was developed by the NHS Institute for Innovation and Improvement Program through review of management literature and input from their improvement experts, senior administrative and clinical leaders to identify over 100 factors considered important for sustaining change [37]. These factors were ranked in a series of focus groups involving 250 NHS staff and health care experts, which led to 10 sustainability factors relating to three domains that are important in sustaining change in healthcare (process, staff, organization) [37]: *Process* explores the benefits of the “change” beyond helping patients (e.g., does the change reduce waste or avoid duplication?), the credibility of the benefits (e.g., do staff believe in the benefits?), adaptability of the improved process (e.g., does the change continue to meet ongoing needs effectively?), and the effectiveness of the system to monitor progress (e.g., is there a feedback system to reinforce benefits and progress and initiate new or further action?); *Staff* explores the involvement and training of staff to sustain the process (e.g., do staff play a rpart in the innovation, design and implementation of the change?), staff behaviours toward sustaining the change (e.g., are staff encouraged and able to express their ideas regularly and is their input taken into consideration?), senior leadership and clinical leadership engagement and support (e.g., are the senior/clinic leaders trusted, influential, respected and believable? Are they involved in the initiative, do they understand it and promote it?); and *Organization* explores the fit with the organization’s strategic aims and culture (e.g., are the goal clearly contributing to the overall organizationa strategic aims?); and infrastructure (e.g., are there policies and procedures supporting the new way of working?). The resulting survey is a self-assessment tool that is designed for teams to identify strengths and weaknesses of the implementation plan and to predict the likelihood of sustainability [37]. Respondents are asked to select from one of 4 response levels for each of the 10 sustainability factors (across the 3 domains). The different levels represent what the respondent perceives as the best fit with their current situation: the highest level represents the most favourable sustainability perspectives, the lowest level represents the least favourable perspective, and the remaining two levels are in the middle. The survey accompanies a sustainability guide, which offers practical advice on achieving sustained use of the initiative by providing the means for teams to address identified challenges and prompt discussion and action to address them, particularly across factors with maximum potential for improvement (i.e., largest difference between identified score and maximum potential sustainability score). The process is aimed at raising *early* awareness of sustainability challenges, and the opportunity for teams to iteratively address these challenges to optimize the new initiative’s potential for impact [37].

#### Population and recruitment

We used a non-probability sampling strategy whereby the NHS survey was administered in May 2014 to all members of the Health TAPESTRY team who were actively involved in the development, implementation and pilot evaluation of the intervention (*n* = 38). The team comprised the following stakeholder groups: *Scientific leads* (*n* = 11): *Research staff* (*n* = 7), *Program staff* (*n* = 5) *Clinic staff* (*n* = 12) and, *Volunteer organization* (*n* = 3) (Additional file 1). An email invitation was sent to Health TAPESTRY team members with the purpose of the survey, and instructions on how to complete it. We excluded recruitment of volunteers because they were not directly involved in the development of Health TAPESTRY. To enhance response rates, we used Dillman’s strategy (3 follow-up reminders approximately two weeks apart) [38].

#### Data collection and analysis

Each member of the team independently completed the sustainability survey on an electronic worksheet developed by NHS (Excel file). The survey represents a diagnostic scoring system designed to bring about the “conversation” around potential barriers using guidance for sustainability [37]. A sustainability score of >55 represents a “reason to be optimistic” (i.e., above the threshold of what is considered a potentially sustainable intervention); and a score of <45 suggests that some action needs to be taken to “increase the likelihood that the improvement initiative will sustain” [37]. Analysis involved a quantitative assessment of the mean scores generated by each respondent’s survey. We aggregated individual mean scores to generate an overall mean team score and corresponding bar graph to highlight scores across each of the 3 sustainability domains and its subdomains. We calculated the largest difference between actual scores and maximum potential score (a pre-specified maximum potential score built into the NHS survey scoring system) to identify best potential for improvement across sustainability factors. Aggregated team results were shared amongst the Health TAPESTRY team during a *Scientific Leads* meeting in June 2014 to discuss the sustainability factors identified as having the most potential for improvement (i.e., greatest difference between sustainability score and maximum potential score).

#### Outcomes and outputs

Primary outcomes were mean team sustainability score and factors across three domains with the greatest potential for improvement. Outputs were a description of the factors influencing sustainability of the intervention and consensus-based suggestions by the Health TAPESTRY team for addressing identified sustainability barriers.

### PHASE 2: Qualitative telephone interviews

To gain a more in-depth understanding of the factors that influence the implementation, sustainability and scalability potential of the Health TAPESTRY intervention, we conducted semi-structured telephone interviews with selected members of the Health TAPESTRY team between July and September 2014.

#### Population and recruitment

We used a purposive sampling strategy to recruit 3–5 Health TAPESTRY team members from stakeholder groups that participated in PHASE 1 survey (*n* = 42): *Scientific leads* (*n* = 11); *Research staff* (*n* = 7): *Program staff* (*n* = 5): *Clinic staff* (*n* = 12): and *Volunteer organization* (*n* = 3). We also included representation from the *Volunteers* providing direct service to clients (*n* = 4). Recruitment involved sending an email invitation describing the purpose of the qualitative interviews to team members. To increase our response rates, we also added a question at the conclusion of the NHS survey (PHASE 1) to invite respondents to participate in qualitative interviews.

#### Interview guide development

We developed an interview guide using findings from the NHS survey as well as the Theoretical Domains Framework [39], which is a useful approach to inform questions that best elicit responses on behaviour change domains. Questions were grouped into three categories: participants’ role in Health TAPESTRY, their general perceptions of the determinants of its sustainability and scalability (barriers and facilitators), and suggestions for overcoming potential barriers. Two experienced moderators conducted 30–60-min interviews (Additional file 2).

#### Data analysis

Interviews were audio recorded and transcribed verbatim. Transcripts were imported into qualitative analysis software (i.e. NVivo 10.0), which was used by two independent reviewers to conduct a multi-step thematic analysis [40]. This involved the reviewers independently reading transcripts and creating an initial list of codes. The reviewers developed a codebook through consensus-based discussions; the codebook was further refined and pilot tested using two interview transcripts. Inter-rater agreement between reviewers was calculated using the Kappa coefficient in NVivo. After each discussion about a discrepancy, one researcher made all appropriate changes to the coding file until a Kappa coefficient of ≥0.8 was achieved for each individual code/transcript combination. The transcript data were divided into five groups, which were coded in sequential rounds using the steps described above. After the first two rounds of coding (*n* = 10 transcripts), the number of discrepancies between the two reviewers decreased substantially, so the remaining three rounds of coding (*n* = 15 transcripts) involved a modified coding approach where one reviewer coded all transcripts and the second coder verified coding on one randomly selected audit transcript per round. Inter-rater reliability was computed for the audit transcript and if any discrepancies arose, the reviewers discussed and resolved them until the Kappa coefficient of ≥0.8 was achieved. Once all data were coded, a series of team meetings was held to discuss the main themes of the data.

#### Outcomes and outputs

Perceptions of stakeholders of the determinants influencing the sustainability and scalability of the Health TAPESTRY intervention. We generated a table of identified barriers and facilitators according to identified themes and mapped the recommendations (as informed by respondents) to address barriers. This output was aimed to help the team make informed decisions about modifying the Health TAPESTRY intervention to maximize its potential for impact.

### Integration of quantitative and qualitative results

To facilitate the integration of data from PHASE 1 quantitative survey with PHASE 2 qualitative interviews, a between-method-data triangulation technique entitled the meta-matrix, was applied [41]. The meta-matrix technique allows for secondary-level triangulation, whereby quantitative and qualitative data are analysed individually, followed by plotting commonalities between them into a matrix. This allows a strong visual anchor to recognize patterns in the data and to recognise confirming/corroborating, elaborating/expanding and identifying contradicting/discrepant views between data types [41]. This technique was employed to allow for a more complete understanding of the sustainability and scalability potential of the Health TAPESTRY intervention. For example, we mapped NHS sustainability domains/sub-domains to the factors influencing sustainability that emerged from qualitative findings.

## Results

Table 1 shows the demographic characteristics of the NHS survey and qualitative interview participants. Of 38 and 42 Health TAPESTRY team members who were invited to participate in PHASE 1 and PHASE 2, respectively, 20 individuals completed the NHS survey (53% response rate) and 25 individuals completed the qualitative interviews (60% response rate). Participation across the 2 studies was representative of the wide range of stakeholder groups involved in the development and pilot testing of Health TAPESTRY (Table 1).

**Table 1:** Demographic characteristics of participants in the NHS sustainability survey and qualitative telephone interviews

Stakeholder group	NHS Sustainability survey (*n* = 20)		Qualitative interviews (*n* = 25)	
	Number invited	Number participated (%)	Number invited	Number participated (%)
Clinic staff	12	5 (42%)	12	5 (42%)
Program staff	5	4 (80%)	5	3 (60%)
Research staff	7	4 (57%)	7	4 (57%)
Scientific Leads	11	5 (45%)	11	7 (64%)
Volunteer organization	3	2 (67%)	3	2 (67%)
Volunteers	-	-	4	4 (100%)

### PHASE 1: NHS sustainability survey

**Fig. 1:**
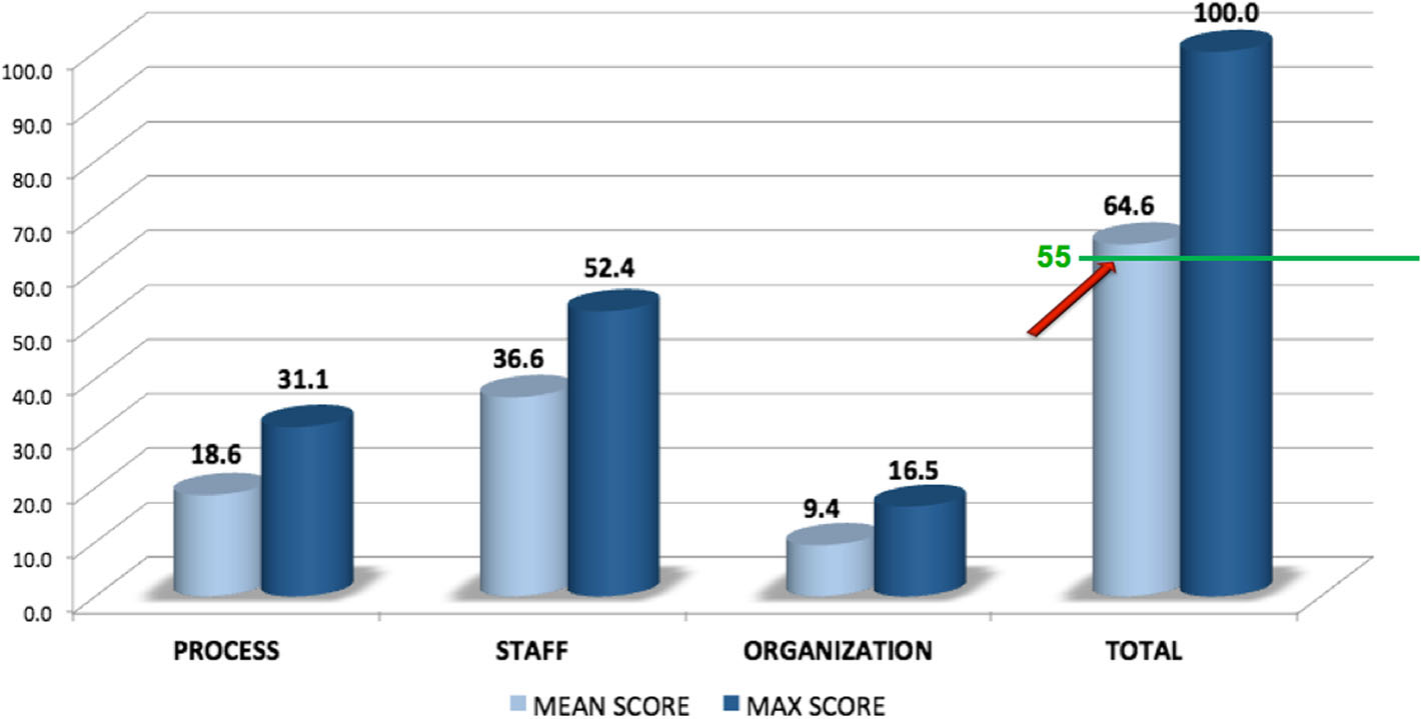
Overall mean sustainability score across the three sustainability domains of the NHS sustainability survey

**Fig. 2:**
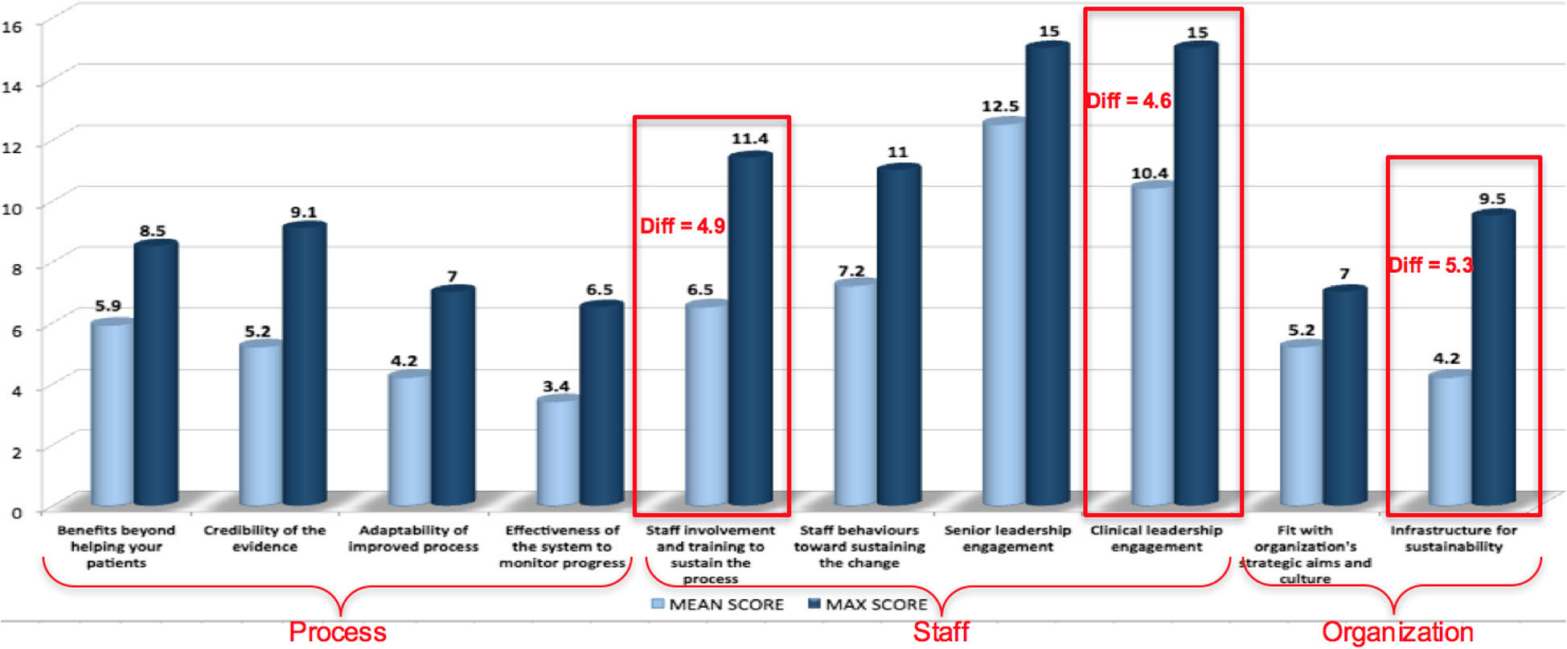
Best opportunities for improvement across the three domains of the NHS sustainability model

Figure 1 shows the mean sustainability score for each of the three sustainability domains of the NHS model (process, staff and organization). The total mean sustainability score was 64.6 (range 22.8 to 96.9), and 70% of survey participants reported a score ≥ 55 (i.e., above the threshold of what is considered a potentially sustainable intervention and “reason to be optimistic”). Figure 2 shows the mean scores for each of the 10 sustainability factors highlighting the difference between team mean score and maximum potential score. The best opportunities for improvement were in the *Staff* domain pertaining to the factors: “staff involvement and training to sustain the process” and “clinical leadership engagement”; and in the *Organization* domain pertaining to the factor: “infrastructure for sustainability”.

### PHASE 2: Qualitative interviews

Sustainability and scalability determinants of Health TAPESTRY emerged across two major dimensions: I) Health TAPESTRY operations, and II) Health TAPESTRY intervention. We defined “Health TAPESTRY operations” as any factor related to activities undertaken by the central Health TAPESTRY developer team (scientific leads, research staff, and program staff) to support the development and implementation of Health TAPESTRY, and the processes by which the program and its team members accomplished this. Examples of activities were developing technological components of the intervention, conducting research studies to understand and evaluate Health TAPESTRY, and coordinating the communication between members of the developer team so they work as a cohesive unit. We defined “Health TAPESTRY intervention” as any factor associated with the intervention and its specific elements (i.e., patient home visits by volunteers to collect information on health goals and chronic disease risks, automated transmission of data to primary care inter-professional team using tablet devices, the Health TAPESTRY report outlining an action plan in response to health goals and risks, and patient access to the PHR). Tables 2 and 3 summarizes the sustainability and scalability determinants of Health TAPESTRY across these two dimensions (operations and intervention) matched with actionable recommendations as suggested by interview informants. We also mapped the NHS sustainability model domains and related factor level questions to identified determinants that the Health TAPESTRY team can consider as a starting point to address identified challenges. A more detailed description of our data is in Additional files 3 and 4.

**Table 2:** Factors that influence the sustainability and scalability of Health TAPESTRY operations^a^ across identified themes; and questions that can be considered to address barriers according to the National Health Service (NHS) Sustainability model^b^

Theme	Summary of sub-themes by challenges and facilitators *Challenges are described in the context of the Health TAPESTRY developer team*	Suggested recommendations (as informed by participants)	NHS sustainability model^b^ factor level
Sustainability			
Program complexity	Challenges: • Health TAPESTRY large and complex with many different facets of research, so there is arge amounts of data to process and interpret. • Health TAPESTRY’s focus and purpose is unclear in terms of how all the pieces fit together, and everything moves slowly due to the large number of people who are involved • Technology development process is difficult because data gathering tools and the application are constantly changing	• Synchronize all the parts of Health TAPESTRY • Make sure that various components and timelines fit together well to achieve program goals	Organization: • Fit with organization’s strategic aims and culture • Infrastructure for sustainability
			Process: • Adaptability of improved process
Program coordination & communication	• Gaps amongst the team to delegate responsibilities to move the project along • Gaps amongst the team in certain skill sets • Difficulties with adhering to and communicating deadlines and timelines • Getting everyone to attend the same meetings Facilitators: • The team is enthusiastic and has excitement and passion in regards to program goals • Scientific leads are experienced and well connected • Program management/research team efficient and motivated	• Expand the central Health TAPESTRY team • Identify the skills and knowledge that are needed internally to fill the gaps • Be transparent in the decision making processes • Provide the Health TAPESTRY developer team with a contact person who can relay their involvement and contribution • Provide progress notes or a weekly update to be distributed to all Health TAPESTRY developer team members	Organization: • Infrastructure for sustainability
			Staff: • Staff involvement and training to sustain the process^a^
Resource capacity	Challenges: • Lack of adequate human resources pertaining to the Health TAPESTRY developer team (too few people doing the administrative tasks to oversee the project, and no one to manage the sub-studies being conducted by scientific leads) • Funding the Health TAPESTRY in general; not being able to secure more funding • Managing pulls and priorities from many organizations for funding within Health TAPESTRY	• Have an intermediary person that will facilitate communication between the two co-Leads and the team and between the volunteers and the Leads • Bring in support on the IT side • Investigate additional research opportunities and consider applying for funding for these; and to evaluate small components of Health TAPESTRY where there is great uncertainty or to make it work better	Organization: • Infrastructure for sustainability
			Process: • Adaptability of improved process
Stakeholder buy-in	Challenges: • The team is diverse, encompassing individuals from a broad range of disciplines and organizations • The inter-professional team has not been involved or considered as part of the central Health TAPESTRY developer team	• Ensure mutual benefits between the project and the clinical and content experts involved continue to exist • Invite the inter-professional team to be more involved in discussions, particularly to assess the best way clinicians could respond to the Health TAPESTRY report data	Organization: • Fit with organization’s strategic aims and culture
Scalability			
Adoption potential	Challenges • It is unclear how data will be shared between sites if they have different hardware (i.e., tablets) and software (i.e., operating system) than pilot site, which may impair optimal functioning of the health tapestry application to create customized reports	• Create an application that will allow Health TAPESTRY to be accessed through any operating system and hardware device (i.e. laptop, tablet, desktop) • Automate the Health TAPESTRY reports to create custom reports depending on the needs of different sites	Process: • Adaptability of improved process^a^ • Effectiveness of the system to monitor progress Organization: • Infrastructure for sustainability Fit with the organization’s strategic aims and culture
	• Unclear how to keep the program consistent through the different sites and provinces that will adopt it • Unclear how program values will be communicated consistently across sites	• Make sure that Health TAPESTRY communicates the same messages as intended by allowing the flexibility for change but keeping it consistent enough that it is still Health TAPESTRY • Present Health TAPESTRY as a flexible approach with some of it key pillars, but understand that it might be implemented in different ways	
	• If the implementation plan is too rigid then Health TAPESTRY might become less scalable • Currently, there is no implementation strategy in place that can be given to adopter sites about issues such as customizability and fit, key learnings from the pilot site, and the ability to monitor intervention use	• Develop an online implementation guide/manual/protocol to give to adopter sites that provides practical information on not just what to do but how to do it (e.g., how to recruit and train volunteers, how to overcome risk management, who should be involved at each step)	
	• There is no process in place for how to nurture adopter sites which do not have the benefit of close contact with the Health TAPESTRY developer team	• Provide an avenue for Health TAPESTRY site to compare their experiences and share their successes and lessons learned • Have someone from the Health TAPESTRY developer team that can trouble shoot and coordinate at other sites initially until they are set up • Have the pilot site provide implementation outreach to new sites	
Adopter site characteristics	Challenges: • Scaling up will be challenging for adopter sites that don’t have the same understanding of how Health TAPESTRY works • It is unknown how Health TAPESTRY will function in culturally diverse populations (e.g. language barriers including the translation of outcome measures)	• Make sure that adopter sites have a complete understanding of Health TAPESTRY and the community in which it will be implemented • Adopter sites should also be made aware that the partnerships that have been built within the pilot site may look very different in expansion sites	Organization: • Infrastructure for sustainability
Readiness to scale	Challenge: • Health TAPESTRY is not ready to be scaled up or sustained in other jurisdictions	• Work out the systems issues in the most supportive environment first (i.e., Hamilton site) to see if and how it works and then scale up • Develop an implementation plan with adopter sites by reflecting on success and lessons learned at the pilot site, and assess how applicable these learnings are to the new site	Process: • Credibility of the evidence • Adaptability of improved process
Resource capacity	Challenge: • Scaling up will be challenging for adopter sites that are not research intensive (i.e., capacity, skills, knowledge)	• Convey to adopter sites that the goal of Health TAPESTRY is not to replace or take over existing processes but to help individuals/organization identify which of its core components are valuable and fit within their setting (i.e., volunteers, the use of a PHR, the use of an interprofessional team, and support from a system navigator)	Organization: • Infrastructure for sustainability
Undetermined efficacy of Health TAP	Challenge: • Lack of demonstration of Health TAPESTRY’s effectiveness and cost-effectiveness	• Generate evidence of effectiveness and cost-effectiveness • Ensure that new knowledge and evidence are disseminated, and to share early results about successes and lesson learned broadly	Process: • Lack of demonstration Credibility of the evidence

^a^Health TAPESTRY operations refers to intervention support elements provided by the central developer team members, which includes the scientific leads, research staff, and program staff. Examples of activities the central team performs includes developing technological components of the intervention, conducting research studies to understand the impact of the intervention, and coordinating communication between the scientific leads, research staff, and program staff so they work as a cohesive unit

^b^Adapted from: Maher L, Gustafson D, Evans A. NHS Sustainability Model. NHS Institute for Innovation and Improvement; 2010. Available at: www.institute.nhs.uk/sustainability

**Table 3:** Factors that influence the sustainability and scalability of the Health TAPESTRY intervention^a,b^ across identified themes; and questions that can be considered by intervention team to address barriers according to the National Health Service (NHS) Sustainability model^c^

Theme	Summary of sub-themes by challenges and facilitators *Challenges are described in the context of specific stakeholder groups*	Suggested recommendations (as informed by participants)	NHS sustainability model^c^ factor level
Sustainability			
Program complexity	Inter-professional team challenges: • Regulations for changing coding in the EMR • Health TAPESTRY (Health TAP) report provides too much information, which could overload clinicians • Potential for clinicians to misinterpret data generated by volunteers during client visits Volunteer team challenges: • How Health TAP and volunteers fit in with existing programs (e.g., Health Links)	• No suggestions were provided	Process: • Adaptability of improved process
			Organization: • Infrastructure for sustainability
Program coordination and comnication	Inter-professional team challenges: • No process in place for how clinicians will use the Health TAP report (e.g., who will take care of the patient once the report is generated, what resource are available to support activities as suggested by the report, and the fear that the report will get lost among other incoming electronic paperwork) • Functionality of the data transmission from iPads to inter-professional team, and how clinicians will recognize that it is a Health TAP report • Interpretation of the Health TAP report by non-clinicians	• Clarifying role and expectations about the report and outline correct health care responses for report findings • \Adjust care processes so that Health TAP becomes part of the what the inter-professional team are already doing	Process: • Benefits beyond helping patients • Credibility of the evidence
	Volunteer team challenges • Increasing number of clients enrolled may make it more difficult to sustain the volunteer pool • Availability of students or younger volunteers (e.g., during exam time and when school is not in session over the summer months) • Coordinating or scheduling the visits and volunteers (e.g., to be able to match volunteers with clients that live near them) • Involvement of a well-established volunteer community organization • Evaluating volunteer competency for appropriately administering data gathering tools	• Keep the volunteer numbers reflective of the program growth • Start recruiting student volunteers at the beginning of the semester and early in their studies to maximize their availability & keep them longer • Have ongoing support for volunteers and volunteer coordinators (keep them updated so they know the details of the visit in advance) • Consider how to prepare volunteers for unknown situations (e.g., hoarding, bed bugs, safety in the client environment)	Process: • Adaptability of improved process^s,b^
			Organization: • Infrastructure for sustainability
			Staff: • Staff involvement and training to sustain the process • Senior leadership engagement
Resource capacity	Inter-professional team challenges: • Managing resources as the number of clients involved increases • Feasibility of administering the many data tools during volunteer-client visits • Not having proper IT requirements during technology development • Resources to upload information into the PHR • Feasibility of Health TAP report (burden on clinicians’ time and workflow to read patient files; how the report fits in their workflow; the effort to use the report; the time it takes away from regular work) • Setting (i.e., the report requires physical space to discuss with colleagues) • Lack of provider skills and knowledge or the supports to manage patients identified as at risk • Bringing Health TAP into an already busy primary care context Volunteer team challenges: • Funding volunteer program since these organizations are largely non-profit • Lack of adequate human resources to recruit enough volunteers, and enough volunteer coordination support to sustain the volunteer pool Client challenges • Health TAP may become too much during the visit (too many questions and data tools)	• Provide support to primary care to manage issues and to incorporate Health TAP report data in a way that integrates its elements into what is already being done at the clinics • Look at opportunity costs (e.g., are the volunteer visits increasing the visits to health care providers, and are these truly necessary) • Make sure that the client visits are accessible or not too far for volunteers • Have a point person for volunteers who would brief them about expectations and to provide positive feedback when they do well	Organization: • Infrastructure for sustainability Process: • Adaptability of improved process^a,b^
Stakeholder buy-in	Inter-professional team facilitators: • The inter-professional team staff are enthusiastic, innovative, and open-minded Inter-professional team challenges: • Slow adoption by clinics and the inter-professional team • Success is dependent on clinicians’ willingness to follow-up on Health TAP reports • Clinical relevance of data gathering tools • Opportunity for inter-professional staff to customize questions in data tools • Competing priorities in primary care to be able to screen patients for Health TAP • Inter-professional team research fatigue Client (patient) challenges: • Lack of buy-in, and feeling uncomfortable with technology • May not believe the value of Health TAP (i.e., the right approach for their health care) • Discomfort with data being shared by many people at the primary care practice • May not always be receptive of volunteers as part of Health TAP • Poor uptake of the PHR by clients • Research fatigue Community agency challenges: • Community engagement (i.e. involving and linking to community organizations that fit with Health TAP’s goals) Volunteer challenges: • \Keeping volunteers engaged and satisfied	• It would be important to integrate Health TAP within the current clinic infrastructure • Ensure that data/data tools are clinically relevant and provide room for adaptation to meet the needs of the inter-professional team • Effectiveness of Health TAP needs to be demonstated and shared (early wins and successes) so people appreciate its potential to benefit patients • Provide assistance to clients in how to access and use the PHR • Persuade other stakeholders in the system (e.g., government, other 3rd party payers, patient advocacy groups, seniors groups) that there are advantages to Health TAP • Foster strong relationships with relevant community organizations early in the implementation process • Engage these organisations in updates and program progress, and provide information on how they can be involved	Process: • Adaptability of improved process (i.e., Health TAP) • Credibility of the evidence • Benefits beyond helping patients Staff: • Staff (i.e., clinicians) involvement and training to sustain Health TAP • Staff (i.e., clients) attitude toward sustaining the change (Health TAP) Organization: • Infrastructure for sustainability • Fit with organization’s strategic aims and culture
Scalability			
Adopter site characteristics	Inter-professional team challenges: • Recognize that partnerships that were built in the pilot site may not resemble partnerships made at adopter sites • There may be varying levels of motivation and interest in Health TAP in other environments • Adopter sites may not know or understand their own environment or jurisdiction • It might be difficult to apply or implement Health TAP in settings without a PHR, a different PHR,if the concept of a PHR is very new, and in paper-based primary care settings • Bringing new ideas and a new program into an already existing culture	• Assess the infrastructure, needs and resources that are already available at adopter sites • Understand the current capacity of adopter sites, and assess how it fits within Health TAP • Recognize that other communities are typically very different or have a different culture • There should be cultural awareness • Have dedicate resources to facilitate program uptake • It will take time to get to know adopter environment and how their system works	Organization: • Fit with organization’s strategic aims and culture Infrastructure for sustainability
Stakeholder buy-in	Inter-professional team challenges: • Inter-professional team may not understand the value added with the Health TAP report Volunteer team challenges: • Keeping volunteers engaged and satisfied as their sense of belonging (as part of Health TAP) may decrease as the program expands	• Display the relative advantage of the Health TAP report to primary care physicians (e.g., no extra time on the part of the clinician to do things) • Persuade other stakeholders in the system (e.g., government, other 3rd party payers, patient advocacy groups, seniors groups) that there are advantages to Health TAP • Facilitate buy-in through employing credible and respected champions in local primary care practices	Process: Credibility of the evidence
			Staff: • Staff attitude toward sustaining the change Staff involvement and training to sustain the process^a,b^
Resource capacity	Inter-professional team challenges: • In private or solo practices, Health TAP may be perceived as overwhelming • The capacity of physicians in other settings to address the problems identified by the Health TAP report • Solo practices may lack the human resources to handle the information generated by Health TAP (e.g., getting volunteers to gather data, processing the reports, and following-up with patients) • Lack of funding at new sites to implement technological requirements of Health TAP (e.g. tablet devices; automated transmission of data to primary care) • Health TAP may not work for family health teams unless they are linked with volunteer and community resources to coordinate volunteers	• Engage with people and organizations outside of the health realm that can support solo practitioners • Map out how Health TAP can move into a smaller community with just a family physician and their nurse • Consider including Health TAP as part of the primary care visit • Have the physician and nurse work together to follow-up with clients • Promote group medical visits • Train a lay person that could deliver educational sessions to a group of patients • Create an IT application that will allow Health TAP to be accessed through any operating system and hardware device	Organization • Infrastructure for sustainability • Fit with organization’s strategic aims and culture Process: Adaptability of improved process
	Volunteer team challenges • Lack of resources in rural settings to coordinate the volunteer component of Health TAP (e.g., lack of access to and recruiting volunteers, lack of transportation of volunteers, lack of a person/organization who can train and support the volunteers) • Ensuring quality of volunteer-client visits as volume increases (i.e., if number of clients expands as program grows)	• Keep volunteer numbers reflective of the expanding client numbers • Find enough skilled people to serve as volunteers • Expand the scope of volunteers (i.e. don’t rely so heavily on students) to consider clients’ relatives, friends or neighbours • Build another category of volunteers called peer support volunteers, who can be trained on the Health TAP approach • Understand the volunteer’s unique settings and adapt volunteer expectations and training accordingly • Ensure the quality of volunteer training is equivalent across the different trainers	
	Community challenges: • The recognition that remote areas may not have the same community resources, supports and manpower to address clients’ needs identified through Health TAP report Other challenges: •Feasibility of adapting Health TAP to other countries with different health systems	• Assess what community services are available and how these systems work at the adopter site • Link family health teams to existing health care programs	

^a^Health TAPESTRY intervention refers to factors that are associated with the intervention and its elements, and the implementation efforts provided by the interprofessional clinical team, community organizations (e.g. CCAC, meals on wheels), volunteer agency, volunteers, and patients. Examples of activities the implementation team performs includes recruiting volunteers, conducting patient home visits, assessing the Health TAPESTRY report, and developing an action plan to help patients meet their health goals

^b^For the NHS sustainability factor: Adaptability of improved process, “process” is referring to the Health TAPESTRY intervention

^c^Adapted from: Maher L, Gustafson D, Evans A. NHS Sustainability Model. NHS Institute for Innovation and Improvement; 2010. Available at: www.institute.nhs.uk/sustainability

#### I. Health TAPESTRY operations

Table 2 summarizes the determinants across Health TAPESTRY operations. We identified four themes related to sustainability, and five themes related to scalability:

##### Sustainability

***Program complexity***: Health TAPESTRY was perceived as large and complex with many different facets of research, which will result in substantial amounts of data to process and interpret. Health TAPESTRY’s focus and purpose was also perceived as unclear in terms of how all the pieces fit together, and described as “everything moving slowly” due to the large number of people who are involved. They suggested synchronizing all the pieces of Health TAPESTRY, to make sure that various components and timelines fit together to reach goals, and to have a better balance in the number of people involved across stakeholder groups. ***Program coordination and communication*** *:* Health TAPESTRY was described as having experienced Scientific Leads that were well connected to relevant clinical and content experts, and were enthusiastic and knowledgeable to meet program goals. Identified gaps were to not delegating responsibilities, certain skill sets, variation in team involvement, communication between leads, research, IT, volunteers, and the community. Recommendations were to expand the central Health TAPESTRY team, provide training, be transparent in the decision making processes, and provide progress notes or weekly updates distributed to the whole team. ***Resource capacity:*** There was concern that the central Health TAPSTRY team may not be able to carry out administrative tasks to oversee the project and manage the sub-studies being conducted, and the long-term funding of the program. Recommendations were to designate a person who would function as an intermediary to facilitate communication between team members, to build resource capacity for IT, and to investigate additional research opportunities to generate additional funding. ***Stakeholder buy-in:*** Informants felt that it was important to maintain buy-in from a broad range of disciplines and organizations and to ensure that the inter-professional team is considered as part of the central Health TAPESTRY developer team. They suggested more involvement of this group in discussions, particularly in terms of how best clinicians could respond to the Health TAPESTRY report data, and to highlight the mutual benefits between the project and the clinical and content experts.

##### Scalability

***Adoption potential*** **:** Health TAPESTRY was perceived to have potential for scale-up in a range of different implementation contexts, but only if implementation plans are not too rigid. Scalability challenges were related to how data would be shared between sites if the adopter site has different hardware (e.g., tablet device) and software (e.g., operating system), and how to keep Health TAPESTRY consistent across different adopter sites/provinces. Suggestions were to develop an online implementation guide to provide practical information on how to recruit and train volunteers, how to overcome risk management, and who should be involved at each step. ***Adopter site characteristics:*** Scaling up was perceived to be a challenge for sites that don’t have the same understanding of how Health TAPESTRY works as the pilot site, and how it might function in culturally diverse populations including language barriers. Suggestions were to make sure that adopter sites have a complete understanding of Health TAPESTRY and the community in which it will be implemented, and to understand that the partnerships that have been built within the pilot site may look very different at their site. ***Readiness to scale-up:*** Many informants indicated that Health TAPESTRY is not yet ready to be scaled up or sustained in other jurisdictions, but recommended sharing learnings from the pilot site, to think about what aspects of the scalability need to be planned, and to work out the systems issues in the most supportive environment first. ***Resource capacity:*** Scaling up will be challenging for adopter sites that lack research capacity. Respondents indicated that it is important to convey that the goal of Health TAPESTRY is not to replace or take over existing processes but to help identify which core components are valuable and fit within the adopter setting. ***Undetermined program efficacy:*** A major perceived scalability barrier was the currently undetermined effectiveness and cost-effectiveness of Health TAPESTRY. Although they recognized that this work is currently underway, respondents highlighted the importance of sharing this knowledge and evidence with adopter sites so they can see what has worked, and where changes might need to be made to optimize success.

#### II. Health TAPESTRY Intervention

Table 3 summarizes the determinants across the dimension of the Health TAPESTRY intervention. We identified four themes related to, and three themes related to scalability:

##### Sustainability

***Program complexity:*** Health TAPESTRY is complex and has many moving parts including IT maintenance, PHR information, regulations for changing coding in the EMR, data collection and update, and integrating Health TAPESTRY within new and existing health care programs (e.g., HealthLinks). ***Program coordination and communication:*** Clinic team challenges were a lack of a process for when and how to use the Health TAPESTRY report by providers, who should take care of the patient once the report is generated, lack of information on what resources are available to support activities suggested by the report, and that the report might get lost among other incoming electronic documents. Suggestions were to clarify specific roles and responsibilities on the report, to clearly indicate the expected and correct health care responses, and to adjust care processes so that Health TAPESTRY becomes part of what the clinic team are already doing. At the level of the volunteer program, challenges involved the difficulty with sustaining the volunteer pool with increasing number of enrolled clients, the availability of student or young volunteers, involvement of a well-established volunteer community organization, and the lack of support for coordinating and scheduling client visits. Suggestions were to keep the volunteer numbers reflective of the expanding client numbers, to provide on-going support and updates to volunteers/coordinators on the overall program, to provide constructive feedback about expectations and performance, and to ensure that volunteers are prepared for unknown situations. ***Resource capacity:*** Challenges experienced by the clinic team were not having enough resources to keep up with the increased number of clients enrolled in Health TAPESTRY, recruiting enough volunteers, and obtaining sustainable funding. Suggestions were to provide additional support to primary care, consider Health TAPESTRY elements that fit into existing workflows, and to consider opportunity costs. Although the technology behind Health TAPESTRY and its integration (i.e., the automated generation of the report) were described by respondents as innovative, it was perceived as requiring a large amount of resources. It was suggested that a plan is needed that takes into consideration the burden on providers’ time and workflow. ***Stakeholder buy-in*** **:** The inter-professional team was described as enthusiastic, innovative, and open-minded about Health TAPESTRY, but client recruitment was perceived as dependent on clinician buy-in or willingness to review and follow-up on Health TAPESTRY reports. Other factors influencing buy-in were clinical relevance of the data gathering tools, the ability to have input into customizing questions, having competing priorities within primary care, the lack of time and processes for screening patients for inclusion, and the potential for provider research fatigue. Clients’ buy-in were influenced by their comfort level with technology, perception of how the program can add to their healthcare experience, comfort with the clinic team having access to their data, and their reception to volunteers. Community level buy-in was related to fostering and maintaining a relationship with community organizations. Respondents suggested engaging community partners in updates and program progress, providing information on how they can be involved, and for the Health TAPESTRY team to demonstrate early wins to benefit patients and the system.

##### Scalability

***Adopter site characteristics:*** Adopter sites need to understand how their environment works, to recognize that other communities are typically very different or have a different culture, and that partnerships that were built in the pilot site may not resemble their own partnerships. Suggestions to facilitate adoption were to get to know the infrastructure, needs and resources that are available at the adopter site including what works and what community services are available, how is it different, and how Health TAPESTRY might fit into it. Respondents felt that it is important for adopter sites to have a full understanding of Health TAPESTRY at the onset of implementation so they can better understand how it might fit into their context, and to understand that it will not replace existing processes. The goal is to help individuals/organizations to identify which core components of the program fit with their needs and setting.

***Resource capacity:*** Respondents were concerned that due to resource constrains experienced by many private, solo, and paper-based primary care practices, Health TAPESTRY may be perceived as overwhelming or difficult to scale up. These practices may lack the volunteer capacity to gather the data and the physicians to address identified problems. Suggestions were to engaging people and organizations outside of the health realm that can support solo practitioners, and to consider Health TAPESTRY as part of the primary care visit. At the level of volunteers, challenges were related to the lack of resources in rural settings to coordinate volunteers (i.e., access to and their transportation), and to train and support them. Additionally, Health TAPESTRY may not work in family health teams that are not linked with volunteer and communication resources. Another identified challenge was how to ensure the quality of volunteer-client visits, particularly as the program expands. Suggestions were to expand the scope of volunteers beyond students such as to consider peer support volunteers (e.g., clients’ relatives, friends, or neighbours). ***Stakeholder buy-in:*** It is important to obtain buy-in from the expansion site’s clinic team to ensure that they view Health TAPESTRY as value added. Respondents felt that it was important to show the relative advantage of Health TAPESTRY including improved care for patients, and to persuade other stakeholders in the system such as government and patient advocacy groups of these advantages. Having credible and respected champions in the local primary care practices was also identified as important. At the level of the volunteer team, informants reflected on the pilot stage when this group was smaller and had a stronger sense of community, which facilitated buy-in and engagement from the volunteers. Respondents feared that as Health TAPESTRY expands with an increased number of volunteers, their sense of belonging to the program may be diminished, lead to decreased commitment to the program or feeling under-recognized or under-appreciated.

## Discussion

**Fig. 3:**
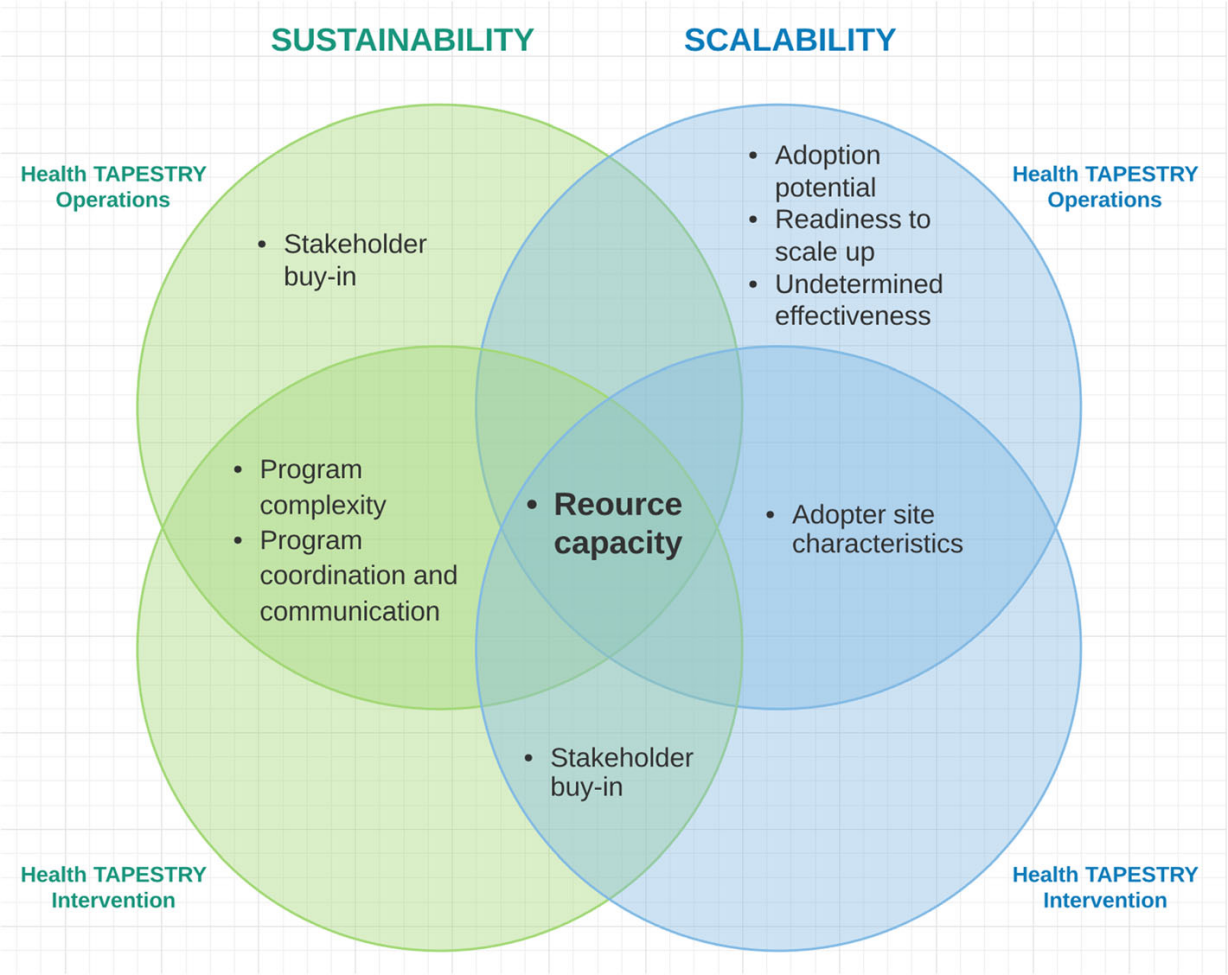
Factors of Health TAPESTRY operations and intervention that were found to influence sustainability and scalability

Our mixed-methods study identified factors that influence the sustainability and scalability of the Health TAPESTRY intervention from the perspective of the team that was actively involved in its development and pilot testing. Our PHASE 1 survey findings helped us to focus a discussion amongst the Health TAPESTRY team about anticipated sustainability challenges, and informed the interview guide that was used for a more in-depth understanding of specific sustainability and scalability determinants. In PHASE 2, our qualitative study of telephone interviews with 25 team members revealed that the sustainability of Health TAPESTRY was influenced by its complexity, coordination and communication, resource capacity, and stakeholder buy-in; while the scalability of Health TAPESTRY was influenced by its adoption potential, adopter site characteristics, readiness to scale-up, resource capacity, stakeholder buy-in, and undetermined efficacy (Fig. 3). These findings helped us to create actionable recommendations (as indicated by informants) that were matched with specific sustainability and scalability challenges. Overall, we found that the resulting themes across the different factor levels of the NHS sustainability model were consistently spread across all three domains (process, staff, organization). The recommendations suggested by informants combined with team discussions (generated by standardized factor-matched questions in the NHS sustainability model) can be used to optimize an intervention such as Health TAPESTRY thereby increasing its potential for impact.

Our overall findings showed that resource capacity appears to be an important factor influencing the sustainability and scalability of Health TAPESTRY both in terms of its operations as well as the intervention itself and its components (Fig. 3). To sustain Health TAPESTRY, informants suggested designating a person who could facilitate communication between team members to oversee the project, carry out administrative tasks, build resource capacity for IT, and to generate additional funding. To ensure that clinic teams have enough resources to keep up with the demand of Health TAPESTRY (i.e., increased number of clients and volunteers), respondents suggested providing additional support to primary care, and to consider only those elements of Health TAPESTRY that fit into existing workflows and provider time, particularly in terms of overcoming the demands to sustain its technology innovation. To scale up Health TAPESTRY, respondents iterated the importance of conveying that the goal of Health TAPESTRY is not to replace or take over existing processes but to help identify which core components are valuable and fit within the adopter setting. To support resource poor primary care practices (i.e., many private, solo and paper-based practices), respondents suggested engaging people and organizations outside of the health realm, and to consider Health TAPESTRY as part of the primary care visit. At the level of volunteers, suggestions were to expand the scope of volunteers beyond students such as to consider peer support volunteers (e.g., clients’ relatives, friends, or neighbours), particularly in rural settings where access to public transportation may be limited.

The investigation of sustainability is seldom considered in research plans or not considered early enough in the development process to be able to address potential challenges. A related challenge is that there is little guidance on *how* to assess the sustainability of KT interventions. There are frameworks for implementing sustainability interventions and for measuring sustainability [22, 27, 42, 43], but a recent scoping review by Tricco et al. to determine the uptake of such frameworks found no studies that used a framework to consider sustainability of a KT intervention in the context of chronic disease management [29]. Furthermore, very little attention has been paid to studying the sustainability and scale-up and spread of specific health care innovations [44] even though these are the investigations that are needed to inform practice and policy decision making. Of reported efforts to scale-up interventions, most do not consider theory-based, rigorous methods for studying scale-up processes, and have involved mostly “conceptual or descriptive” analyses with a narrow focus [44]. The lack of consideration of sustainability can lead to implementation failure and wasted resources [34–36]. Understanding the determinants of sustainability and taking action to address them involving all knowledge users, has the potential to facilitate implementation efforts of change initiatives, and can help support sustained improvements in health care services and patient outcomes over time.

Our study contributes to advancing the knowledge of systematically investigating the sustainability of complex KT interventions such as Health TAPESTRY. Another strength of our study is that we not only identified potential sustainability and scalability factors, but also attempted to map suggestions and recommendations by respondents to identified factors as well as to the NHS sustainability model, to elucidate what steps may be actionable to overcome them. Individual scores were highly variable (range 22.8–96.9), which is a reflection of the wide range of stakeholders involved in the implementation of Health TAPESTRY. However, the goal of the NHS survey is to derive a team score, which is the level at which we can best reveal opportunities for improvement, to facilitate a common understanding of challenges, and to promote consensus-based solutions to address them. The actionable recommendations are therefore representative of the collective perceptions of the Health TAPESTRY team, making them more relevant for decision making. In fact, informant suggestions were directly applied to make changes to Health TAPESTRY (e.g., to clarify aspects of the automated report; improve volunteer coordination and recruitment) as well as to improve the communication between the various stakeholders involved in Health TAPESTRY (e.g., regular updates via monthly electronic newsletters). Lastly, our findings contribute to the value and feasibility of a structured process related to our study design (i.e., sequential mixed-methods approach) that can be used by teams to iteratively inform change. We suggest that these methods could be applied to any complex health care delivery intervention.

Our study has some limitations. First, we used the NHS sustainability survey, which aims to enable teams to recognize and self-assess key sustainability factors at an early stage of implementation and to prompt discussion and action across domains and sub-domains with the largest potential for improvement [37]. However, some participants found the use of this survey challenging (e.g., the number and understandability of the questions, forced choice statements, and its value in elucidating sustainability barriers). Another study that applied the NHS sustainability survey found similar challenges [22]. However, these challenges may be overcome by providing more instruction to participants on how to complete the survey. Additionally, since the survey was administered to a wide scope of Health TAPESTRY stakeholders, participants who were less familiar with all facets of the intervention and its developmental steps (e.g., volunteer organization) found it difficult to understand and relate the terminology used in the survey to the Health TAPESTRY intervention. Third, the response rate for our survey was moderately low (53%), so our sample and findings may not be representative of the entire implementation team. Furthermore, findings may not be generalizable beyond the sample of Health TAPESTRY-affiliated stakeholder groups who participated in our studies. However, we had a balanced representation of our stakeholder groups across our sample of participants in both phases of our investigation. Fourth, it was a challenge to prioritize within all of the data in terms of clarifying what are the critical factors that could impair the sustainability and scalability of an intervention such as Health TAPESTRY. However, sustainability and scalability are interrelated, and so we suggest that a development team cannot address identified sustainability and scalability factors in isolation. Fifth, our findings could not elucidate which elements need to be consistently applied and how best to balance this with the contextualization that is needed. When research programs are implemented in real-world settings, some degree of adaptation of program components occurs [45–47]. There is some evidence that it can be beneficial to allow some adaptation to local context in order to facilitate buy-in and receptivity of program content [48, 49]. This will be an important consideration when developing an implementation guide for Health TAPESTRY for the purpose of scaling up the approach across Canada. It will also be important to evaluate this process to determine what aspects of the approach should change to maintain fidelity, and which components can allow flexibility without impacting outcomes.

## Conclusions

Our study identified sustainability and scalability determinants of the Health TAPESTRY intervention that can be used by the Health TAPESTRY team to optimize its potential for impact. Next steps will involve using findings to inform a guide to facilitate sustainability and scalability of Health TAPESTRY in other jurisdictions considering its adoption. Our findings build on the limited current knowledge of sustainability, and advances KT science related to the sustainability and scalability of KT interventions.

## Additional files

Additional file 1: Characteristics of Health TAPESTRY team stakeholder groups. This table shows the breakdown of Health TAPESTRY team member types, the number in each category, and their definition. (DOCX 16 kb)

Additional file 2: Qualitative telephone interview semi-structured questionnaire. This is the interview guide that was used for the qualitative telephone interviews. It shows the questions, and how they are mapped to the theoretical domains framework (TDF) and their constructs. (DOCX 21 kb)

Additional file 3: Identified challenges to the sustainability of Health TAPESTRY and recommendations to address them as indicated by respondents. This table shows the identified sustainability challenges by theme and sub-theme that emerged from qualitative telephone interviews as well as the recommendations to overcome the barrier (as suggested by respondents) accompanied by relevant quotes. (DOCX 43 kb)

Additional file 4: Identified challenges to the scalability of Health TAPESTRY and recommendations to address them. This table shows the identified scalability challenges by theme and sub-theme that emerged from qualitative telephone interviews as well as the recommendations to overcome the barrier (as suggested by respondents) accompanied by relevant quotes. (DOCX 51 kb)

## Abbreviations

EMR: Electronic medical record
FHT: Family health team
Health TAPESTRY: Teams advancing patient experience: strengthening quality
KT: Knowledge translation
NHS: National health service
PHR: Personal health record
