# Supplementary material for: Sustainability and scalability of a volunteer-based primary care intervention (Health TAPESTRY): a mixed-methods analysis
Source: BMC Health Serv Res. 2017 Aug 1;17:514. doi: 10.1186/s12913-017-2468-9 (PMC5540508; doi:10.1186/s12913-017-2468-9)
Supplement: Supplementary file 1 — Characteristics of Health TAPESTRY team stakeholder groups. This table shows the breakdown of Health TAPESTRY team member types, the number in each category, and their definition. (DOCX 16 kb) [file 12913_2017_2468_MOESM1_ESM.docx]

**Appendix A**

Characteristics of Health TAPESTRY team stakeholder groups

| **Stakeholder group** | **n** | **Definition** |
| --- | --- | --- |
| **Health TAPESTRY team** | 38 | - Individuals who were involved in the development, implementation, and pilot evaluation of the HEALTH Tapestry intervention. - The team can be broadly categorized into the _ categories described below. |
| **Scientific leads** | 11 | - Individuals who lead research projects of the Health TAPESTRY program according to their expertise in patient engagement, geriatrics, nursing, interprofessional teams, information technology, health economics, medications, data management, and rehabilitation science. - Individuals who make decisions related to all Health TAPESTRY program components including strategic planning and operationalization; and steer the development, implementation and evaluation plan of Health TAPESTRY. |
| **Research staff** | 7 | - Individuals who are involved in coordinating, administering, and supporting the research across the Health TAPESTRY program. - Individuals who developed outcome assessment and measurement instruments and documented, communicated, managed and analyzed data. |
| **Program staff** | 5 | - Individuals who are involved in supporting the Health TAPESTRY program including administrative and technology support, and volunteer coordination. |
| **Clinic staff** | 12 | - Health care providers (family physicians, nurses, pharmacists), unit directors, managers and other allied health professionals (social workers, dietitians, occupational health professionals, and system navigators) who have provided consultation to the program development and who provide clinical support at the McMaster Family Health team (FHT). |
| **Volunteer organization** | 3 | - Members of the volunteer organization involved in the Health TAPESTRY program (Shalom Village). |
