# Supplementary material for: Sustainability and scalability of a volunteer-based primary care intervention (Health TAPESTRY): a mixed-methods analysis
Source: BMC Health Serv Res. 2017 Aug 1;17:514. doi: 10.1186/s12913-017-2468-9 (PMC5540508; doi:10.1186/s12913-017-2468-9)
Supplement: Supplementary file 2 — Qualitative telephone interview semi-structured questionnaire. This is the interview guide that was used for the qualitative telephone interviews. It shows the questions, and how they are mapped to the theoretical domains framework (TDF) and their constructs. (DOCX 21 kb) [file 12913_2017_2468_MOESM2_ESM.docx]

**Appendix B**

Qualitative telephone interview semi-structured questionnaire

| **Interview questions** | **TDF Domain (definition)** | **TDF domain constructs** |
| --- | --- | --- |
| 1. **Please describe your current role in Health TAPESTRY**    - *What has been your involvement in Health TAPESTRY?*    - *What specific tasks [things] are you involved in / responsible for?* 2. **In terms of your role, what do you still want to accomplish within Health TAPESTRY?**     - *How do you see your future role in Health TAPESTRY?* | **Social/Professional Role and Identity**  *A coherent set of behaviours and displayed personal qualities of an individual in a social or work setting* | Professional identify; Professional role; Social identify; Identity; Professional boundaries; Professional confidence; Group identify; Leadership; Organisational commitment |
|  | **Beliefs about Capabilities**  *Acceptance of the truth, reality, or validity about an ability, talent, or facility that a person can put to constructive use* | Self-confidence; Perceived competence; Self-efficacy; Perceived behavioural control; Beliefs; Self-esteem; Empowerment; Professional confidence |
|  | **Goals**  *Mental representations of outcomes or end states that an individual wants to achieve* | Goals (distal/proximal); Goal priority; Goal/target setting; Goals (autonomous/controlled); Action planning; Implementation intention |
|  | **Intentions**  *A conscious decision to perform a behaviour or a resolve to act in a certain way* | Stability of intentions; Stages of change model; Transtheoretical model and stages of change |
|  | **Reinforcement**  *Increasing the probability of a response by arranging a dependent relationship, or contingency, between the response and a given stimulus* | Rewards (proximal/distal, valued/not valued, probable/improbable); Incentives; Punishment; Consequents; Reinforcement; Contingencies; Sanctions |
| 1. **From your perspective, what are the specific outcomes** *[end results]* **that Health TAPESTRY will be able to achieve?**  - *What are the benefits as a result of Health TAPESTRY* - *This could be in terms of patients, the team, community, etc.* - *How optimistic are you that these outcomes will be achieved?*  1. **What do you think are the barriers to achieving the outcomes you described?** | **Beliefs about consequences**  *Acceptance of the truth, reality, or validity about outcomes of a behaviour in a given situation* | Beliefs; Outcome expectancies; Characteristics of outcome expectancies; Anticipated regret; Consequents |
|  | **Optimism**  *The confidence that things will happen for the best or that desired goals will be attained* | Optimism; Pessimism; Unrealistic optimism; Identity |
|  | **Emotion**  *A complex reaction pattern, involving experiential, behavioural, and physiological elements, by which the individual attempts to deal with a personally significant matter or event* | Fear; Anxiety; Affect; Stress; Depression; Positive/negative affect; Burn-out |
| 1. **What is your impression of the team’s skills and training in terms of sustaining Health TAPESTRY** *[to make sure it keeps going]***?**  - *Are there specific skills or training that you think still need to be addressed right now to facilitate the sustainability / scalability of Health TAPESTRY?*  1. **Are there particular groups within the team that you think SHOULD be more involved in Health TAPESTRY?** | **Skills**  *An ability or proficiency acquired through practice* | Skills; Skills development; Competence; Ability; Interpersonal skills; Practice; Skill assessment |
|  | **Knowledge**  *An awareness of the existence of something* | Knowledge (including knowledge of condition/scientific rationale); Procedural knowledge; Knowledge of task environment |
|  | **Social influences**  *Those interpersonal processes that can cause individual to change their thoughts, feelings, or behaviours* | Social pressure; Social norms; Group conformity; Social comparisons; Group norms; Social support; Power; Intergroup conflict; Alienation; Group identify; Modelling |
| 1. **Overall, what do you think are the facilitators of Health TAPESTRY?** *[what works well / what are the strengths of Health TAPESTRY?]* 2. **Overall, what do you think are the barriers of Health TAPESTRY?** *[what does NOT work well in Health TAPESTRY?]* 3. **Do you have any suggestions on how we could improve Health TAPESTRY** 4. **What do you think are the barriers to the sustainability of Health TAPESTRY** *[to make sure it keeps going]***?**  - *Is the infrastructure there to facilitate sustainability? (e.g., are there enough facilities an equipment to support Health TAPESTRY?...are there policies and procedures in place?)* - *At what point would it become difficult to sustain Health TAPESTRY (i.e., what are the boundaries of what is possible)?*  1. **What specifically do you think we need to think about or do RIGHT NOW to facilitate the sustainability of Health TAPESTRY** *[to keep it going]***?**  - *How do you see Health TAPESTRY keeping going?*  1. **How do you see Health TAPESTRY being scaled up** *[spreading it wider]***?**  - *What do you think might influence the scaling up of Health TAPESTRY* - *How would this work in rural parts of Ontario / other provinces*  1. **In what situations might it be difficult to scale up Health TAPESTRY? What are the boundaries of what might be possible?** 2. **Do you think we are ready to scale up Health TAPESTRY? Why/Why not?** | **Environmental context and resources**  *Any circumstance of a person’s situation or environment that discourages or encourages the development of skills and abilities, independence, social competence, and adaptive behaviour* | Environmental stressors; Resources / material resources; Organisational culture / climate; Salient events / critical incidents; Person x environment interaction; Barriers and facilitators |
|  | **Memory, Attention and Decision Processes**  *The ability to retain information, focus selectively on aspects of the environment and choose between two or more alternatives* | Memory; Attention; Attention control; Decision making; Cognitive overload/tiredness |
|  | **Behavioural regulation**  *Anything aimed at managing or changing objectively observed or measured actions* | Self-monitoring; Breaking habit; Action planning |
| 1. **Do you have anything else to add? …do you have any questions?** | NA | NA |
