# Supplementary material for: Sustainability and scalability of a volunteer-based primary care intervention (Health TAPESTRY): a mixed-methods analysis
Source: BMC Health Serv Res. 2017 Aug 1;17:514. doi: 10.1186/s12913-017-2468-9 (PMC5540508; doi:10.1186/s12913-017-2468-9)
Supplement: Supplementary file 3 — Identified challenges to the sustainability of Health TAPESTRY and recommendations to address them as indicated by respondents. This table shows the identified sustainability challenges by theme and sub-theme that emerged from qualitative telephone interviews as well as the recommendations to overcome the barrier (as suggested by respondents) accompanied by relevant quotes. (DOCX 43 kb) [file 12913_2017_2468_MOESM3_ESM.docx]

**Appendix C**

Identified challenges to the sustainability of Health TAPESTRY and recommendations to address them as indicated by respondents

| **Theme** | | | | **Sub-theme [participant number]** | **Recommendations to overcome challenge: *relevant quotes*** |
| --- | --- | --- | --- | --- | --- |
| **1. Health TAPESTRY intervention features** | | | | | |
| **Data gathering tools** | | | | | |
|  | | Clinical relevance | | Not clinically relevant [I-6] | - Need to think about what criteria should be used to select data gathering tools for Health TAPESTRY: *“whatever the results, it should be clinically relevant and be comprehensible by the clinician”* [I-6] - *“The tools should mean something for a clinician, to be more clinically applicable”* [I-6] |
|  |  |  |  | Some of the tools are more beneficial for the research side but not for the clinical setting [I-6] | - The first priority should be the clinical setting; research is secondary – *“We are researching based on what benefits are being given to primary care, but if they are not getting benefit and we are only performing research than that doesn’t make sense”* [I-6] |
|  |  | Feasibility | | Volume of data gathering tools [I-23] | - |
|  |  | Flexibility | | No opportunity for clinicians to augment questions in data gathering tools (to make more customizable) [I-23] | - Adaptability and flexibility are the key things that would make Health TAPESTRY sustainable [I-6] |
| **Health TAPESTRY Application** | | | | | |
|  | | Buy-in | | There could be some resistance on the part of the elderly patients who may be uncomfortable with technology or using computers, and may feel that this [Health TAPESTRY] is not the right kind of approach to receive their care [I-2] | - |
|  |  | Data sharing | | Discomfort with the fact that patient health record is being shared, and so many people can contribute to it [I-2] | - |
|  |  | Process of technology development | | Not having the proper IT requirements from the team in advance can create a lot of extra work [I-4] | Provide clear direction of IT requirements in terms of what is needed for the project: *“It’s easy for people to say, ok make this change, but from the programming point of view, that little change might take up to a week. We try the best from what we know, but these requirements change all the time and they create a lot of work on our end” [I-4]* |
|  |  |  |  | Application is constantly changing [I-4] |  |
|  |  | Programming | | Getting information into the PHR [I-19] | - |
|  |  |  |  | Programming is a challenge because of regulations for changing coding in the EMR [I-16] | - |
|  |  | Uptake | | There’s probably a small majority of seniors who will not access the electronic application and PHR on their own [I-8] | - Need to provide help for seniors to be able to access and use the PHR: *“So if the plan is to keep volunteers going into the homes of seniors, then yes I can see it continuing [Health TAPESTRY], but I don’t if they don’t have help and if seniors are then responsible on their own to access it [PHR] and identify various issues through that, I don’t know if that would continue”* [I-8] |
| **Health TAPESTRY Report** | | | | | |
|  | | Feasibility of use | | | |
|  |  |  | *Burden on physicians’ time and workflow* | Physicians don't have the time to read client files; Additional time and effort needed by physicians to use the report [I-3, I-5] | - There are some things that can be discussed with patients during the Health TAPESTRY visit that would help family physicians focus on some other things such as accessing food services or meals on wheels if there was a nutritional risk that was identified: *“… they [Volunteers] could refer to a community resource such as the Alzheimer’s Society if that client scored really low on cognitive functioning”* [I-17] - Re-engage Volunteers to help collect follow-up information for Physicians: *“The other thing that will probably help is if the volunteer visits collect information, send us a report but people will actually want to know how things went with their patients and if there was any way to re-engage the volunteer, to go back and say, oh your doctor’s actually asked me to come back four months later for a follow-up…how are you doing in terms of your health goals; ‘they sent me back to ask you some questions to see how this is going’, to keep looping back with the patient, that would also be helpful”* [I-24] |
|  |  |  |  | Physicians will need time to figure out where the report fits into their workflow and what information is relevant and useful [I-16] |  |
|  |  |  |  | Report takes time away from physicians’ regular work [I-3] |  |
|  |  |  |  | Report will be one more thing in physicians’ inbox [I-3] |  |
|  |  |  |  | Report requires possibly generating an action plan [I-3] |  |
|  |  |  | *Features* | A lot of information on the Tapestry report [I-3] | - |
|  |  |  | *Functioning* | Initially, reports were generated manually, which is not sustainable [I-4, I-6] | - Reports need to be generated automatically [I-4, I-6] |
|  |  |  |  | It’s not clear that it’s a Health TAPESTRY report until you open it [I-23] | - |
|  |  |  |  | Information collected represents one-way communication only [I-23] | - |
|  |  |  | *Interpretation* | It may be problematic that someone else (other than a Physician) will identify or interpret an issue as urgent/not urgent [I-3, I-17] | - |
|  |  |  | *Setting* | Report requires physical space to discuss with colleagues [I-3] | - |
|  |  | Process of use | | No process in place for what physicians will do with the Tapestry report, how they will use it, who will do what, and how follow-up will happen, [I-3, I-5, I-13, I-25] | - Clarify roles on the report and what the expected and correct health care responses should be: *“…whose roles is what activity and where are the resources coming from”* [I-25] - There needs to be support as Health TAPESTRY reports come in and adjust care processes so it becomes part of what the IP team is doing; Need to constantly make it part of the conversation [I-24] |
|  |  |  |  | Unclear who should take care of the patient once the Report is generated (*“is it the Physician or the Tapestry team?”*) [I-17] |  |
|  |  |  |  | Not clear what resources are available to support activities as suggested by the Report [I-25] |  |
|  |  |  |  | Timing of the communication of the Report between Volunteers and Physicians has not been worked out or a process in place [I-17] | - *“If things are left for two or three days in terms of getting the Report that could put someone at risk for something serious [I-17]* |
|  |  |  |  | Fear that the Report will get lost in the shuffle of other incoming electronic paperwork [I-17] | - |
| **Volunteers** *(as part of the intervention)* | | | | Clients’ reception of volunteers may not always be positive [I-5] | - |
|  |  |  |  | Potential for client misinterpretation of what the volunteer is doing during the visit [I-5] | - |
|  |  |  |  | Not clear how the volunteers sit within groups that are involved in the Health Links experience in the community – will Health TAPESTRY become part of this or a variation of the bigger picture? [I-25] | - |
|  |  |  |  | It’s not clear how the trained volunteers are going to be continually part of Health TAPESTRY and how seniors are going to be responsible for accessing their records through the PHR and contact them that way [I-8] | - |
| **2. Health TAPESTRY Program** | | | | | |
| **COMPLEXITY AND SIZE** | | | | Health TAPESTRY overall is very complex, and there are a large number of components to evaluate, and it’s not always clear how all the pieces fit together or will be executed [I-2, I-3, I-8, I-10, I-11, I-12, I-15, I-16] | - Synchronize all the parts [I-2] |
|  |  |  |  | There are many different facets of research that are going on and going on at the same time, which will only going to increase as the larger trial begins, with more data to process and interpret, and more writing to be done [I-3, I-10, I-11, I-16] |  |
|  |  |  |  | Overlap between all the groups is a challenge [I-12] |  |
|  |  |  |  | Health TAPESTRY is large, and we may not be able to show any significant outcomes at the end of the project (at the end of 2016) as far as healthcare outcomes [I-16] |  |
|  |  |  |  | Health TAPESTRY’s focus and purpose is not clear [I-10] |  |
|  |  |  |  | Large number of people involved in Health TAPESTRY, it’s too large [I-1, I-12, I-14] | - There needs to be a balance of how many people (within the program) are involved in Health TAPESTRY [I-3] |
|  |  |  |  | Everything moves slowly because the team is so large [I-10] |  |
| **EXECUTION AND FUNCTIONING** | | | | | |
|  | | **Patient level** | | | |
|  |  |  | *Adoption* | At what point does Health TAPESTRY become too much for clients during the visit (too many questions) where it may become ineffective? [I-13] | - |
|  |  |  | *Perceived value* | Patients may have the perception that Health TAPESTRY is not the right approach to health care [I-2] | - |
|  |  |  |  | Patients are not always so clear on the purpose of Health TAPESTRY [I-11] | - |
|  |  |  | *Research fatigue* | Patients may have research fatigue [I-3] | - |
|  |  |  | *Use of technology* | Discomfort in using technology [I-2] | - |
|  |  | **Primary care level** | | | |
|  |  |  | *Adoption* | Adoption of Health TAPESTRY by clinics and interprofessional team [in Hamilton] has been slower than anticipated [I-2, I-16] | - Health TAPESTRY is a program that should live within clinics well after the RCT is done, so it needs to be something that fits for clinics, that is meaningful for them, that provides information that they wouldn’t otherwise have: *“We need to study things in a controlled way to give the bigger learnings – these will impact sustainability in terms of understanding what happens in the RCT, what happens at each site as they begin to take up the Health TAPESTRY approach”* [I-10] |
|  |  |  |  | Change doesn’t come easy for clinicians [I-3] |  |
|  |  |  | *Competing priorities* | Many competing priorities within Primary care [I-3] |  |
|  |  |  |  | Healthcare professionals are overwhelmed with various other initiatives [I-19] |  |
|  |  |  |  | Time and process for screening patients for inclusion was onerous during the pilot [I-17] |  |
|  |  |  |  | Health TAPESTRY involves physicians having to deal with many different clinical areas [I-5] |  |
|  |  |  | *Knowledge and skills* | Physicians may not have the skill set or knowledge to manage seniors identified as at-risk [I-1] |  |
|  |  |  | *Lack of support* | Lack of support for family physicians to manage people who are identified as at-risk [I-1] |  |
|  |  |  | *Nature of setting* | Bringing Tapestry into an already busy clinical context [I-1] |  |
|  |  |  | *Privacy concerns* | Privacy issues if the patient health record is shared by many people [I-2] |  |
|  |  |  | *Research fatigue* | Physicians can have research fatigue [I-3] |  |
|  |  |  | *Use of technology* | Discomfort of clinic staff in using technology [I-2] |  |
|  |  | **Health TAPESTRY team level** | | | |
|  |  |  | Gaps amongst the team in terms of additional skills and delegating responsibilities to move the project along [I-16] | | - Having a more experienced team [I-4] - Adding numbers to the central Health TAPESTRY team [I-16] - Identify the skills and knowledge that are needed internally to fill the large parts of Health TAPESTRY and to help streamline it [I-16] |
|  |  |  | Research team needs to be more involved and proactive [I-3] | |  |
|  |  |  | Research team is weaker on the quantitative analysis and assessment [I-3] | |  |
|  |  | **Volunteer level** | | | |
|  |  |  | *Quality of visits* | If there are too many clients, it may affect or hinder the quality of the volunteer interviews/visits [I-5] | - Keep the volunteer numbers reflective of the expanding client numbers [I-5] - Make sure that the number of clients per volunteer is reasonable: one or two or three clients so that the volunteers don’t have to go to so many visits per week, and that they don’t feel overworked and underappreciated [I-5] |
|  |  |  |  | At what point are we asking too much of volunteers to do during a client visit? [I-13] |  |
|  |  |  |  | Introducing new volunteers to the same patients makes it difficult to establish a rapport with them [I-18] | - Need to have continuity for student volunteers [I-18] - Recruit students earlier in their study years to keep them longer [I-18] |
|  |  |  | *Recruitment* | It’s a challenge to recruit student or younger volunteers [at Hamilton site] because of timing such as exams (at which time they are more busy) and not being around during the summer months. This could be an issue in other sites as well [I-5, I-11] | - Starting recruitment in the beginning of the University semester (September and October) to maximize availability of student volunteers [I-1] |
|  |  |  | *Scheduling and coordination* | Coordinating and scheduling the visits and volunteers; getting patients involved and having people coming into their home and coordinating this long-term [I-20, I-25] | - *“Having someone at various regions that can trouble shoot on site will be initially helpful, whether there is funding or not, someone who can coordinate for the other centers”* [I-1] |
|  |  |  |  | To be able to match volunteers with clients that live near them [I-11] | - *“Make sure the client visit are accessible or not too far for student volunteers because many of them take the bus”* [I-11] |
|  |  |  |  | As client and volunteer ratio increased from the pilot, volunteers were not always being informed of visit schedule (where and when it will take place) [I-5] | - Keep volunteers updated so they know the address of the visit and know the details ahead of the time and with enough time [I-5] |
|  |  |  | *Support* | Not having support for volunteers and volunteer coordinators [I-5, I-19, I-24] | - Volunteers need to have somebody who tells them clearly what the expectations are and also provides positive feedback when they do well: *“Volunteers want to feel useful and if you don’t have someone helping them and encouraging them and making them feel useful, you will have trouble”* [I-24] - Ongoing support for the volunteers is key: *“Someone else going out with the volunteers and actually doing the visits and provide some ongoing kind of support and education is a really important piece”* [I-19] - Support the volunteer coordinator; make sure that the person in charge of the volunteers has the adequate resources to inform the volunteers of important information quickly and efficiently [I-5] [I-5] |
|  |  |  | *Training* | Sustaining a volunteer pool and training may be barriers since this has so far been done with a fairly homogeneous population [I-1] | - There will be an online training site (virtual learning centre) where volunteers will be able to go through each section including a test, which they will have to pass to go on [I-11] - Need to consider how to prepare volunteers for some unknown situations that may not be so great that are happening in the client home (e.g., hoarding, bedbugs, other social issues, safety) [I-13] |
|  | |  |  | Evaluating volunteers’ competency for assessing questionnaires has not been addressed – What should be the minimum requirement for a volunteer? [I-13] |  |
|  |  |  |  | Volunteers have been waiting for a long time to actually receive training and get started since the launch date [RCT] has been pushed by 3 months [I-11] |  |
| **OPERATIONAL AND ADMINISTRATIVE** | | | | | |
|  | | Adherence to deadlines | | Adhering to timelines, and to funder timelines [I-3] | - *“We don’t have the luxury to take our time to answer some of our questions before rolling it out and promoting in other communities” [I-3]* |
|  |  |  |  | Lack of communication to meet deadlines [I-16] |  |
|  |  |  |  | Lack of accountability on meeting deadlines [I-16] |  |
|  |  |  |  | Managing timelines of IT projects and programmers [I-16] |  |
|  |  |  |  | Timelines and RCT launch date keep getting pushed, and thus may lose volunteers in the process [I-11] |  |
|  |  |  |  | Timelines are very tight so adhering to them is a challenge [I-10] |  |
|  |  |  |  | A small team right now, with a lot of things to do which may not be completed on time [I-4] | **-** |
|  |  | Meetings | | Leads meetings are not representative [I-1] | **-** |
|  |  |  |  | Leads meetings always cancelled [I-13; I-22] | **-** |
|  |  |  |  | Not everybody can be at the same meeting at the same time [I-16] | **-** |
|  |  | Other | | Getting ready for the RCT has been slow [I-10] | **-** |
|  |  |  |  | To have enough time to devote to Tapestry [I-3] | **-** |
| **RESOURCES** | | | | | |
|  | | **General** | | Having the resources to keep up with the increased number of patients involved in Health TAPESTRY [I-23] | - Need the resources to be able to accommodate the volume of things that are identified [I-23] - In the clinical setting, need a separate or parallel system to deal with the Health TAPESTRY items or issues and problems that come up for people. It would need to be accommodated into the current schedule or current clinical process [I-23] - In the initial phase of Health TAPESTRY, being able to support primary care in terms of resources to manage the issues identified at the beginning, and then it should reach a steady rate and then it won’t be as hard to stay on top of it [I-23] - The right amount of resourcing will depend on the volume in terms of the number of patients, number of reports, number of volunteers [I-25] - To incorporate Health TAPESTRY in a way that integrates its elements into what is already being done at the clinics [I-25] |
|  |  |  |  | Engaging with clinical settings [I-3] |  |
|  |  | **Human resources** | | | |
|  |  |  | *Central Health TAPESTRY team* | Lack of manpower in general [I-3, I-4, I-8, I-15, I-16, I-17] | - Important resources that are necessary for the pilot phase are research assistants, training the volunteers, and for the personal health record [I-19] - Add more people to divvy up duties [I-8] - Having a more experienced team [I-4] - Adding numbers to the central Health TAPESTRY team [I-16] - Identify the skills and knowledge that are needed internally to fill the large parts of Health TAPESTRY and to help streamline it [I-16] - There needs to be an intermediary between the two co-Leads and the team [I-16] - Health TAPESTRY will need support on the IT side [I-21]; and to have someone dedicated to the server to make sure it is up all the time and to deal with server issues [I-4] - Health TAPESTRY will need support to facilitate communication between the volunteers and leads [I-21] |
|  |  |  |  | Everyone is overworked, overwhelmed with too many responsibilities [I-3, I-8, I-19] |  |
|  |  |  |  | Research team are overworked and stretched [I-3] |  |
|  |  |  |  | Fear of spreading too thin and not doing things well; workload is too much [I-3, I-16] |  |
|  |  |  |  | Since the project got so large, there is going to be more and more data that will need to be processed and interpreted, and more writing to be done [I-3, I-8] |  |
|  |  |  |  | There are only one to two people who do a lot of the administrative tasks to oversee the project [I-17] |  |
|  |  |  |  | There isn't a person to manage the sub-studies being conducted by Scientific Leads [I-16] |  |
|  |  |  |  | There isn't a person with methodology and evaluation experience to run the RCT [I-16] |  |
|  |  |  |  | Fear of losing staff [I-3] |  |
|  |  |  | *Volunteer team* | Spending too much money on staff to administer volunteers will not be unsustainable [I-18] | - Support for volunteers is key to sustainability in terms of the training [I-19] |
|  |  |  |  | Getting the number of volunteers needed. If Health TAPESTRY opens up to more physicians, it will become very overwhelming very quickly [I-17, I-25] |  |
|  |  |  |  | Some people may think that the roles that volunteers take on should be a paid position rather than volunteer position [I-18] |  |
|  |  |  |  | A graduate student may not be there very long [I-18] | - Need to consider the timing of student volunteer recruitment [I-13] |
|  |  |  |  | Recruiting student volunteers over the summer, Christmas time or during exams will be difficult [I-13] |  |
|  |  | **Funding** | | | |
|  |  |  | *Health TAPESTRY program* | Not being able to securing more funding [I-3, I-12, I-16] | - Create a change culture and study how Health TAPESTRY gets embedded in the culture of organizations to ensure that when the funding is not there, when it’s no longer an RCT, that Health TAPESTRY is sustainable [I-1] - See where there are additional research opportunities and consider applying for funding for these [I-2] - It has to be a living, evolving process to allow it to continue to grow, so thinking about where additional funding can come from right now to carry it beyond 2016; “*To keep Health TAPESTRY going will require a whole different level of time and money and commitment and effort”* [I-3] - Seek additional funding to evaluate small components of Health TAPESTRY where there is great uncertainty or to make Health TAPESTRY work better than it is now [I-2]   - *Example 1: To study which methods are optimal to engage patients and what are the impacts on patient engagement with the Health TAPESTRY Application technology [I-2]*   - *Example 2: An intervention to get patients to start using the computer so they can fully participate in Health TAPESTRY* - Have to look at the opportunity costs, and what are the hidden things that are not in dollars and cents [I-16] |
|  |  |  |  | Losing champions to keep Health TAPESTRY moving forward once the funding runs out [I-12] |  |
|  |  |  |  | There are a lot of pulls and priorities from many organizations for funding within Health TAPESTRY [I-1] |  |
|  |  |  |  | Health TAPESTRY is not moving in a way that the funder would want [I-22] |  |
|  |  |  |  | Scientific leads do not have complete control of the budget that has been allocated for their part [I-22] |  |
|  |  |  |  | There may be resistance to Health TAPESTRY because its infrastructure may increase costs [I-2] |  |
|  |  |  | *Volunteer program* | It would be a challenge to sustain a program such as Health TAPESTRY financially for volunteer organizations, which are largely non-profit [I-7, I-13, I-16, I-19] | - When the program relies on volunteers, there needs to be funding to train them, and to have the buy-in [I-7] - Have to look at the opportunity costs, and what are the hidden things that are not in dollars and cents – are the visits by the volunteers increasing the visits to health care providers, and are these truly necessary or unnecessary [I-16] - *“To recruit for that group of people without an advertising budget or without staff to support it outside of their regular role, I can see that would be a challenge. What I can say is that you know being apart of the RCT it certainly has benefits that has been attached to it. And so you know our benefit [volunteer organization] was that we are apart of the RCT so I was able to hire an additional volunteer coordinator”* [I-13] |
| **3. Health TAPESTRY Team** | | | | | |
| **BUY-IN** | | | | | |
|  | | Primary care | | Physicians won’t continue with Health TAPESTRY if they don’t see it as value-added in their primary care setting; expectation that the additional workload will be done without showing that it makes a difference [I-19] [I-3] | - Need to have a continued evaluation and refinement of the intervention, and to demonstrate its effectiveness and usefulness [I-1, I-2, I-12, I-15]: “Hopefully to overcome that we need to demonstrate effectiveness and disseminate the knowledge of whatever the effectiveness is and where changes might need to be made based on the evaluation, and that dissemination is going to be really critical for the practice environment. We have to kind of get them pumped up to be excited about how it worked and to sell it, to market it, assuming we can demonstrate effectiveness. If we can’t demonstrate effectiveness well then the story completely changes”. [I-2] - To show that Health TAPESTRY is useful within the health care system, to show that Health TAPESTRY is an academic initiative, that it’s a sustainable approach [I-15]: *“I understand that we need to increase awareness about an innovation, but we need more than that, we need more than to just talk about it or tell people about it, we need to actually demonstrate some early wins, you know, some, some really novel sort of successes or differences of the program, so that people can really appreciate some of the potential of it, even though it may not be fully evaluated or fully formed”* [I-2] - Need to demonstrate early wins and some really novel successes or differences of Health TAPESTRY so that people really appreciate its potential [I-2]: *“Show that a group of GPS just really like it, so a quick win, where the relative advantage can be very helpful in getting people excited”* [I-2] - Need to monitor Health TAPESTRY to make sure it is being delivered in the right way and that it’s meeting certain targets (moving from the research world to the quality assurance world) [I-2] - Need to increase awareness of the innovation [Health TAPESTRY] [I-2] - Need to disseminate the knowledge of the effectiveness not just for primary care [I-2] - Need to show government what’s in it for them and what will the results of Health TAPESTRY will be for any given patient [I-7] - Showing that Health TAPESTRY can keep people living in their homes longer and healthier and that this program will not strain the system will help keep Health TAPESTRY sustained and to keep the money coming in [I-7] - To show that there will be a benefit to patients [I-7, I-19] - To show that there will be a benefit to the FHTs, to have the information about the difference that Health TAPESTRY will make [I-19] - To show in the long term that Health TAPESTRY is a national model that actually changes care [I-1] - The goal of Health TAPESTRY is to keep people in their homes longer and healthier, which are things that will help not strain the system for older adults – if this can be proven, it will help it sustainable and to keep money coming in [I-7] - Show that Health TAPESTRY is sustainable as a whole different layer in the healthcare system [I-18] |
|  |  |  |  | How to keep so many people thinking about aging in this slightly different way [Health TAPESTRY] or for healthcare professionals to change their practice in a way that will stick [I-3] |  |
|  |  |  |  |  |  |
|  |  |  |  | Lack of primary care champion for volunteers and volunteer program [I-12] | - Need to have the champions in the local primary care practices who are credible and respected [I-12] |
|  |  | Volunteer organizations | | Need to also have buy-in from volunteer organizations [I-2] | - “It’s not just for the primary care, but it’s going to have to be for the volunteer organizations who have been involved as well as any community service organizations we get involved. We have to kind of get them pumped up to be excited about how it worked and to sell it, to market it, assuming we can demonstrate effectiveness. If we can’t demonstrate effectiveness well then the story completely changes”. [I-2] |
| **COMMUNICATION** | | | | | |
|  | Among and between teams | | | Lack of updates on what is going (progress on Health TAPESTRY development and research) and what has already been completed [I-12, I-13, I-22] | - *“Need to be very clear about what everyone is doing, not just the ones working tightly together or the few who are doing things that nobody knows about”* [I-22] - There needs to be transparency to decision making, and being very clear what the activities will be for the next few months and to unpack the decision making process around those activities [I-14] - To making sure that the larger Health TAPESTRY team has a contact person who they can relay their involvement and contribution; and to make sure that the larger Health TAPESTRY team are included in any discussion that would happen [I-14] - Provide progress notes or a weekly update to be distributed to the whole team [I-5, I-12, I-22]:   - *“If we can’t have meetings (which is extremely challenging to find meeting times for these big teams to get together), it’s just murder, and so the only other way would be through maybe writing up short progress notes to be submitted for distribution to the entire team. I know it’s more work and the last thing I need is more work, but I think it would be a valuable thing to do just so people know and we could see where we’re maybe overlapping”.* [I-12]   - *“But I suppose just to have an update, this is what we have done, like an update sent to community volunteers and whoever else is part of the overarching team, just updating ok so far in Tapestry we’ve done this and our next goals are this, perhaps just to keep everyone in the loop.* [I-5]   - *“I think having a lot of small pieces, communication always helps. Even like a weekly update, just ok well we’ve got 12 pilot patients and our first two volunteers have gone out. Little things like that, this is our first tiny little report about the statistics and out of ten people interviewed eight of them are considered frail, it would be nice. But right now I’m not seeing that happening… maybe communication is a good place to start to make sure everybody is aware of what’s going on and then giving people the opportunity to have input”* [I-22] - The Leads will have to consider other stakeholders and make them aware of what Health TAPESTRY is trying to do and to consider some of their issues [I-2]   - *“I was able to set up regular communications with the project coordinator for Tap as a whole. And so that was absolute, that is essential, so that would occur on a weekly basis, and then as far as connecting with other,”* [I-21] |
|  |  |  |  | Lack of interaction between the research and the core program management team [I-3] |  |
|  |  |  |  | Decisions are made by the research team which scientific leads are not aware of [I-22] |  |
|  |  |  |  | Research team needs clarification on roles [I-3] |  |
|  |  |  |  | Clinicians should be acknowledging as being part of the research team [I-23] |  |
|  |  |  |  | There is a lot of grey zones in the communication flow [I-16] |  |
|  |  |  |  | Not knowing whether communication between volunteers and Scientific Leads should sit with the primary care practices or located in the research or academic environment [I-21] |  |
|  |  |  |  | Lack of communication between volunteers and community organizations [I-19] |  |
|  |  |  |  | Feeling left out with no support from Health TAPESTRY [I-22] |  |
|  |  |  |  | Lack of awareness of next steps of Health TAPESTRY [I-22] |  |
|  | For project execution | | | Lack of communication to meet deadlines [I-16] | - |
|  |  |  |  | Volunteers are not being informed of when and where client visits will take place [I-5] | - |
| **ENGAGEMENT** | | | | | |
|  | | Primary care groups | | Clinician groups should be more involved [I-3, I-6] | - Need a lot of really strong partnerships between multiple systems (the volunteer system, the IT system, the community organization system, and the primary care teams). Partnerships with all of these groups are vital [I-12] - Research done on partnerships shows that you’ve got to have buy-in for the goal that you’re hoping to achieve. People need to make sure that they are all on board with consensus on “this is what our goal is: keep older adults at home as long as possible”. [I-12] - Need strong partnerships between multiple systems: the volunteers, IT, community organization, and primary care teams [I-12]: *“So you’ve got to have those champions in the local primary care practices, who are credible and respected, that can help get that buy-in for them to participate.”* [I-12] - Need not only to study us [Health TAPESTRY team] but to also do some work of Knowledge Translation [I-15] |
|  |  |  |  | Interprofessional teams are moving slower than volunteer and technology [I-16] |  |
|  |  | Community organizations | | Community engagement is the least developed of the 4 arms of Tapestry [I-16] |  |
|  |  |  |  | Loss of connection with Public Health in Hamilton as a collaborator [I-3] |  |
|  |  |  |  | Not enough involvement or linkages with community agencies and organizations (CCHC, Alzheimer’s society, Home care, OT/PT, Personal support workers, shopping support, yard work) [I-19] |  |
|  |  | Knowledge translation group | | The Knowledge Translation group is not as involved in Health TAPESTRY as they should be, to be actually part of the team [I-15] |  |
|  |  | Volunteer organization | | Frequent changes in timelines have significantly impacted the relationship between volunteer organizations and some student volunteers [I-13] |  |
