# Supplementary material for: Sustainability and scalability of a volunteer-based primary care intervention (Health TAPESTRY): a mixed-methods analysis
Source: BMC Health Serv Res. 2017 Aug 1;17:514. doi: 10.1186/s12913-017-2468-9 (PMC5540508; doi:10.1186/s12913-017-2468-9)
Supplement: Supplementary file 4 — Identified challenges to the scalability of Health TAPESTRY and recommendations to address them. This table shows the identified scalability challenges by theme and sub-theme that emerged from qualitative telephone interviews as well as the recommendations to overcome the barrier (as suggested by respondents) accompanied by relevant quotes. (DOCX 51 kb) [file 12913_2017_2468_MOESM4_ESM.docx]

**Appendix D**

Identified challenges to the scalability of Health TAPESTRY and recommendations to address them

| **Challenge themes and subthemes** | | | | | | **Recommendations to overcome challenge: *relevant quotes*** |
| --- | --- | --- | --- | --- | --- | --- |
| **1. Buy-in** | | | | | | |
| **Demonstration of effectiveness** | | | | | | |
|  | Program effectiveness | | | Haven’t shown if the program actually works (does it actually allow for better care for older adults?) [I-11] | | - Need to generate evidence of effectiveness and cost-effectiveness to foster scaling up [I-2, I-15] - Being able to show that 1 or 1 ½ years after the RCT is done that this was worth the investment [I-10] - Need to show that there is evidence of some potentially positive outcomes for clients and for the healthcare team [I-10, I-19]:   - *“Once people see that there are benefits, it will help with their willingness to scale up to use it in different areas”* [I-10]   - *“ If Health TAPESTRY allows for better care of older adults or complex patients then clinicians will adopt this approach”* [I-19] - Make it all easy to get to, and support sites by evidence [I-15]   Dissemination of knowledge from effectiveness evaluations:   - Information about early results should be shared with the Hamilton FHT sites (McMaster and Stonechurch) so they can see how it has worked, what information is given to the team and whether it’s been helpful – this will allow more physicians to jump on board [I-9] - Need to disseminate the knowledge of whatever the effectiveness is and where changes might need to be made based on the evaluation [RCT] [I-12] - Dissemination will be critical for the practice environment (primary care) as well as for the volunteer organizations who have been involved, and the community service organizations we will get involved – they need to be excited about how well it worked and to sell it and market it [I-12] - Roll it out at other sites the way it was done in Hamilton, but provide them with lessons learned [I-12] |
|  | Cost effectiveness | | | Haven’t shown whether the program is cost effective [I-11, I-12] | |  |
| **Partnerships** | | | | | | |
|  | Lack of involvement of an interprofessional (IP) team as part of the core Health TAPESTRY decision makers as the project expands [I-24] | | | | | - Need to involve the interprofessional team who looks at the Health TAPESTRY reports, and make sure they are involved in how they respond to the reports [I-24] |
|  | Lack of partner or collaborator involvement for spread | | | | | - There should be mutual benefit between the project itself and the experts that are involved (e.g., discipline-specific or methods) then there are greater chances to spread [I-6] |
|  | The partnerships that have been built up within the Hamilton site may be a very different situation elsewhere [I-1] | | | | |  |
| **Perceived value** | | | | | | |
|  | Primary care | | | Scalability will be difficult if a primary care office doesn’t see Health TAPESTRY as value added [I-1] | | - Showing the relative advantage of no extra time on the part of the clinician to do things [I-2] - Need to persuade other stakeholders (i.e., government, other 3rd party payers, patient advocacy groups, seniors groups) in the system that there are advantages to Health TAPESTRY or to increase their awareness of Health TAPESTRY otherwise it won’t be possible to move it to something that becomes readily adopted [I-2] - Clinicians need to feel that the care of their patients has improved or it makes their life easier [I-1] - Need to get champions in the local primary care practices who are credible and respected to help get that buy-in for them to participate [I-12] |
|  |  |  |  | Lack of buy-in from physicians, without which recruitment will be difficult during expansion; getting physicians and clinics involved [I-17, I-11] | |  |
|  | Stakeholders | | | Sustainability will depend on its perceived value by stakeholders [I-10] | |  |
| **Readiness to scale up** | | | | | | |
|  | Readiness in general | | | Health TAPESTRY is not ready to be scaled up; the Hamilton model may not be sustainable in other jurisdictions [I-3, I-4, I-5, I-10, I-12, I-13, I-16, I-17, I-19] | | - It takes a lot to have research personnel who is able to insert themselves in the team; take learnings from the Hamilton site to share with other sites [I-10] - Need to think about what are the aspects of the scalability approach that has to be planned now vs. what is available [I-15] - Try and work out the systems issues in the most supportive environment to see if it works and then try and scale it from there [I-12] |
|  | Readiness at the local level | | | Tapestry is not ready at the local level and already jumping into expansion [I-3] | | - Need to show that Health TAPESTRY is successful at the local level before spreading [I-3, I-17] - Implement and test Health TAPESTRY in private practices first before thinking about incorporating it community or province-wide; Need to invest in local physicians or people that are in the local community that can pilot the program, which will make it easier for recruitment – having physicians that are more engaged and involved and work together [I-17] - Roll out to other sites using a similar strategy as Hamilton: *“Start with a pilot on a smaller scale because they may not necessarily be ready to roll out on a bigger scale nor should they before understanding and evaluating the problems”* [I-12] |
| **2. Characteristics of adopter sites** | | | | | | |
| **Environment of adopter site** | | | | | | |
|  | **Understanding the environment** | | | | | |
|  |  | Available resources | | Not knowing or understanding the other jurisdictions or environments [I-13] | | - Get to know the infrastructure, needs and resources that are already available at other sites, what is working, and how Health TAPESTRY might fit into it, would the concept be the same or different, and how is it different [I-6];   - *“Unless we know them, we are not going to make it sustainable in those regions. For example, the concept of interprofessional team is very common here in Hamilton but not very common in other regions like Saskatchewan or other remote areas, right? So how Tapestry is going to work in that region, whether the interpreofessional team would be the same, the structure of the interprofessional team, the concept would be the same, or would it be little bit different as compared wo what we see here in Hamilton? So I think there shouldn’t be the knowledge barrier, we should know each and everything about that area before applying Tapestry on that”* [I-6] - Need to know the community services and really know how the system works there [I-6] - It is a lot easier to scale up when you have people that know what the resources are and that they can access those resources [I-17] - Make sure that the other sites have a full understanding of Health TAPESTRY [I-11] |
|  |  | Diversity of the population | | We don’t know how Health TAPESTRY will function in culturally diverse settings [I-16] | | - Recognize that communities are typically very different and have a different culture, so learning about them is key [I-22] - Need to have cultural awareness, and everyone involved in Health TAPESTRY should be cognizant of those subtleties [I-16]: *“Should partner with volunteer organizations or local organizations that are within the different cultural communities and for them to take on the role of a stakeholder to have buy-in – this will support Health TAPESTRY within that community”* [I-16] - Need to accommodate the diversity, and accommodate for aging adults of different cultures and languages to sustain the program and to have it recognized as a national program [I-5]: “The base of our population is very diverse, and it’s important to reflect that in the Health TAPESTRY program” [I-5] |
|  |  |  |  | Health TAPESTRY may not be able to work in settings where language may be a barrier including the translation of measures [I-2, I-5] | |  |
|  |  | Knowledge and skills of physicians | | Capacity of physicians to address the problems identified by Health TAPESTRY [I-17] | | - *“For example, I had one of m patients that actually had come back about a potential risk for elder abuse. And again, in our office, we’re privileged to having people that have experience with that as well as people that know how to ask the right type of questions, and I can foresee that if I were to work by myself in the community, I might not be able to address that properly or address that in the best way because I just don’t know enough about how to manage that, so although it’s good that the volunteers are putting up a lot of things that are necessary to be addressed, but sometimes the doctors don’t have the capacity or the knowledge to actually address them properly”* [I-17] |
|  |  |  |  | Hamilton site is research intensive, and the group is used to participating in different studies and have an understanding of how that works – other sites may not have these advantages [I-10] | |  |
|  |  | Site motivation and interest | | Scale-up will depend on the motivation and interest in Health TAPESTRY [I-3] | | - Successful scalability is going to depend on how motivated and interested adopter sites are in the core pillars of Health TAPESTRY [I-3] - Centers that have the passion or vision will be successful [I-1] |
|  | **Rural settings** | | | | | |
|  |  | Access to and recruitment of volunteers | | Lack of access to volunteers and difficulty of recruiting volunteers in rural areas; particularly student volunteers in areas without College or University [I-5, I-11, I-13] | | - Recognising the transportation available to each volunteer and then planning accordingly, so if a volunteer can only walk to places then maybe don’t send them very far; or limit the type of volunteer that will participate [I-5] - It is important to understand the volunteer’s unique setting and understand the added time they may require to access remote locations [I-5]   - *“A volunteer could be a relative, friend or a neighbour, so it could work that somebody accesses the Health TAPESTRY training to understand what their roles, scope, and responsibilities are, someone that is approved by the primary care physician or nurse practitioner, whomever is delivering primary care who could be associated or they’re going to work together so that relationship could be built” [I-15]* - *“I mean we're lucky here we got university, we got a college...right? I can see that that might not always be so easy. So either this being offering up some flexibility in terms of who the volunteers should, could, would be”* [I-13] |
|  |  |  |  | Lack of volunteer transportation in rural communities and supporting those that don’t drive or live outside of bus routes may make it difficult to do regular visits especially in the winter [I-5, I-11, I-13, I-20] | |  |
|  |  | Community resources | | Remote areas may not have the same community resources (e.g., home care) [I-23] | | - In rural settings, need to engage with people and organizations outside of the health realm that can support solo practitioners. For example, a local library can provide support in terms of printed and electronic resources for patients, and offer space to have educational sessions; pharmacists can also support certain initiatives [I-24] - *“In rural sites, whomever is involved as key stakeholders could get together, and figure out what community resources are available that could be leveraged by making more visible what people need, and developing whatever system is needed within that context – this could include linking to not only what is in the community but through telemedicine resources that are external to that community; it could be linking to tertiary centers not just primary care however that community is organized, and understanding how to leverage these resources”* I-23] |
|  |  | Human resources | | In rural settings there may not be the supports and manpower to address patient needs identified by Health TAPESTRY risk assessment tools [I-17] | | When thinking about expanding, there has to be care pathways that need to be established so that physicians who for example may get an influx of cognitive screens, then they need to know and have the supports on how to manage that in their community [I-17]  *“In urban centres we are a little spoiled because we have people that we can quickly refer to or to call up to get instant advice, but definitely in a rural community where the same issues exist, but the manpower is not there can make it very difficult because of trying to address patient needs and then how to address the patient”* [I-17] |
|  |  |  |  | Rural areas without supports will be a challenge (such as to have a person to train and support volunteers; who provides the volunteer training program) [I-19] | |  |
| **Organization of adopter site** | | | | | | |
|  | **Family Health Teams (FHTs)** | | | | | |
|  |  | Health TAPESTRY in other FHTs may not work without linking with volunteer and community resources to coordinate the volunteers [I-25] | | | | - In the FHT environment, there should be some dedicated resources to facilitate uptake of Health TAPESTRY: *“…there should be volunteers and the community and the resources to coordinate the volunteers”* [I-25] - Health TAPESTRY can be nicely linked into the FHTs as part of the Health Links initiative: *“Health TAPESTRY is nicely aligned with Health Links because many people are building tools and becoming more aware of how to identify complex patients or high end-users of the system – the processes and the tools might not be exactly the same but it might be beneficial in FHTs involved in HealthLinks to connect them and to make sure there is a defined process for when this information comes in and what are the responses to it”* [I-25] |
|  | **Private/solo practices** | | | | | |
|  |  | Feasibility | | A solo physician would find Health TAPESTRY overwhelming [I-24] | | - Need to know the volume of reports that might be coming in [I-25] |
|  |  |  |  | Solo sites may not be used to or have difficulty having to monitor charts or give feedback on things [I-10] | |  |
|  |  | Human Resources | | Scale-up may be possible in the urban and FHT or full service settings where there are resources and there are enough people to make it happen, but this is not sustainable in a private practice where there is no other person except the one physician working [I-8, I-10, I-15, I-17, I-18, I-20] | | - In solo/private practices, people who come in could complete the questionnaire as part of the visit by setting aside an extra half hour while they are there, and then have the physician or other staff look at it later [I-9] - There are very few practices that would have no nurse, so the physician and nurse could work together to follow-up patients; this could be facilitated by providing education and discussion with the nurse: *“I don’t see why the nurse couldn’t be engaged n following up”* [I-24] - It could be set up so that it [Health TAPESTRY] is part of the visit, an extra half hour set aside to give people the Health TAPESTRY questionnaires while they wait at their doctor’s office and then hand it in and have the doctor look at it later – this would allow the continuation of it without having all the people involved that are involved now [in the Hamilton site] [I-9] - There is a potential for group medical visits as a way of overcoming the challenge of solo practices using Health TAPESTRY, where a sub-population in the practice can be identified (based on a topic of interest or importance such as advance care planning) and then arrange for someone to come and meet with that group to talk with them about this [I-24] - Train a lay person that could deliver educational sessions to a group of patients [I-24] |
|  |  |  |  | There isn’t the staff in private practice to handle the information generated by Health TAPESTRY in terms of getting the volunteers to go and ask the questions and processing these questions, and to follow-up [I-9] | |  |
|  |  |  |  | Health TAPESTRY may not be sustainable in a private practice where there is no other person except the one physician working [I-17] | |  |
|  |  |  |  | Scalability will be difficult if a primary care office doesn’t have the nursing support [I-1, I-24, I-25] | |  |
|  |  | Organization of primary care team | | Implementing Tapestry in settings where the organization of primary care or primary health care team is different from pilot site [I-1, I-2]; | | - Need to map out how Health TAPESTRY can move into a smaller community with just a family physician and their nurse in a remote location [I-16] - Need to collaborate with province-level systems and directors in terms of the way primary care is organized to facilitate uptake [I-21]: *“We are working with McMaster family practice and have the directors basically at the table, then that facilitates that scale of implementation and then beyond that you are just looking at bigger structures that would have to be on board in order for it to happen”* [I-21] - Need to know how Health TAPESTRY is going to work in other sites, whether the IP team would be the same, the structure of the IP team, and the whether the overall concept is the same or would it be different [I-6]: |
|  |  |  |  | Scalability will be difficult if a primary care office is not part of a Family Health Team (FHT0 [I-1, I-24, I-25] | |  |
|  |  |  |  | The concept of an IP team may be very new to some sites; independent practitioners don’t necessarily have access to an IP team; The concept of IP team is very common in Hamilton but may not be in other regions [I-6, 24] | |  |
|  |  | Perceived value | | In primary care practices that are not part of a FHT or don’t have the nursing support, unless they [clinicians] see Health TAPESTRY as value-added, they’re not going to see the value for themselves to continue [I-1] | | - *“Unless they [physicians] feel that the care of their patients has improved, or it makes their life easier, Health TAPESTRY might be a commitment that some groups may not decide to take on… so because the real challenge is that we’re implementing a model from a fairly resource rich family health team to very different family practice settings”* [I-1] - *“…if you read information from a Tapestry volunteer who has collected information about one of your patients, and you don’t have a lens that sees that this could be a red flag for something important, this could mean that my patient doesn’t have the financial resources and struggling to actually pay for their medication and therefore not taking it, if you don’t have a lens that says this might be a red flag, some of the information on the Tapestry report could be seen as not useful”* [I-24] |
|  |  |  |  | Solo sites may not perceive the outputs of Health TAPESTRY as useful; may not want to take on the commitment [I-1, I-6, I-24] | |  |
|  | **Resources of adopter sites** | | | | | |
|  |  | General supports | | Health TAPESTRY is a commitment that some groups without the resources may not decide to take on [I-1] | | - Resources will determine the extent to which Health TAPESTRY can be scaled up; Health TAPESTRY will be adaptable by considering the facilities, the services and the resources that they have [I-6, I-16] - Need to know the infrastructure, the resources and all the things that are already there in other regions [I-6] - Resources are needed on all sides; the volunteers, the people who coordinate the volunteers, and the family health team in terms of what is identified through the reports and our ability to respond to them [I-25] - Need to understand the additional resources that are needed to run Health TAPESTRY [I-10] - Make it all easy to get to, and support sites by evidence and support it by infrastructure [I-15] - *“As Health TAPESTRY goes out into other provinces, how will the people currently taking care of this in the Hamilton site (i.e., regular contact with coordinators and other supports to help and walk through issues and answer questions) be able to offer the same level of supports to other sites? – this is a huge undertaking”* [I-8] |
|  |  |  |  | As Health TAPESTRY goes out to different provinces, all the supports that currently exist in the Hamilton sites may not exist there [I-8] | |  |
|  |  | Funding/costs | | Recruitment of volunteers without an advertising budget or staff to support it outside of their regular role would be challenging [I-13] | | - When rolling out a program that would be bigger than the current RCT, it would be good to have the funding settled to be able to have a volunteer coordinator position within the volunteer organization. This would impact on the type of role and the amount of time required from someone [I-13] - In other settings and jurisdictions (e.g., an Indian reserve in Saskatchewan), need to consider who would pay for transportation costs such as cabs for volunteers; and for other things such as police checks, TB tests, parking and tolls and whatever other things that may be needed for volunteers [I-13] - Have to look at the value of Health TAPESTRY and how to look at all its components in terms of the other settings [I-16] - “After the funding is done, how will this [Health TAPESTRY] live on in the clinic so whatever is put in place it needs to fit” [I-10] |
|  |  |  |  | Not every clinic may be able to afford iPads [I-4] | |  |
|  |  |  |  | Health TAPESTRY is turning out to be a full-time role for the volunteer coordinator at the volunteer organization, and it needed the support through the RCT to be able to fund this – not clear how this funding will continue beyond the RCT [I-13] | |  |
|  |  | Human resources | | | | |
|  |  |  | *Support from core TAPESSTRY team* | Level of attention currently received by physicians from Health TAPESTRY staff in terms of letting them know that a Report is coming will likely go away when the program expands [I-17] | | - For Health TAPESTRY to go out to different sites, it will take somebody that looks after the PHR, IT and the App, a local coordinator who looks after the volunteers, quality assurance of the volunteer program, and the communication piece regarding who would take care of the and scientific leads, administrative support, and the aspect of who will actually going to be running the project and who is going to be ultimately, responsible for the project in that province and all the way down [I-8, I-16, I-17, I-19]   - *“I just think that from this team, if there were more physicians involved, I think the amount of attention would go down to each individual practice, but I think that level of attention is necessary to kind of just at the beginning prompt the physicians, like watch out for it you know, because sometimes it matters in terms of the intervening and if things are left for two or three days, that could be two or three days to put someone maybe at risk for something serious [I-17]*   - *“I think if you opened it up to lets say all 20 physicians at our clinic, it’s going to become very overwhelming very quickly, so you would actually need to ramp up a significant number of people to make it work… if you have one person talking to 20 doctors and you have 20 doctors then wanting to get support and arrange home visits or to talk about clarifying what was the screening tool, it can get very complicated. I can see it getting unscreenable in terms of the amount of work for one person. So I think that’s my fear with regards to sustainability and scalability” [I-17]* - Have a case management model for the clinicians in terms of making sure they are following through and they read the reports and they try to work on a path of care for the particular person [I-17] |
|  |  |  |  | The current Health TAPESTRY team will not be physically in the other sites to provide support [I-3] | |  |
|  |  |  |  | Challenge at other sites if there is not enough staff to follow-up and to process the data and reports generated by Health TAPESTRY [I-9] | |  |
|  |  |  |  | The Hamilton site is currently helping with the 4 provinces in which Health TAPESTRY is being rolled out, but this is a huge undertaking as it requires contact with the Hamilton site on a regular basis, walking them through things and answering any questions that they have [I-8] | |  |
|  |  |  |  | Needing to be up in five different sites across Canada by the fall of 2014 will impact on the capacity to do things locally [Hamilton] [I-3] | | *“I worry that we don’t yet have an established program here in Hamilton and at the same time that we are also trying to establish that we also have to be in 4 different parts of Canada, and I think that’s going to spread our team out too thin”* [I-3] |
|  |  |  | *Information technology (IT)* | Every site might have their own survey that they want to add on, which means that they will have to hire their own programmer to create custom reports [I-4] | | - Need to think about whether other sites need their own team to set up their IT or do they want the Hamilton site to host it: *“…currently, the model is that Hamilton will host their version of the APPs for them”* [I-4] |
|  |  |  |  | Not every team will be able to afford a team of IT professionals [I-4] | |  |
|  |  |  | *Primary care team* | Hamilton site is very resourced with many different health care providers. There are other sites that may just have a doctor or nurse, which would make it difficult to implement core concepts of Health TAPESTRY such as system navigation and technology [I-10] | | - |
|  |  |  |  | Some settings may not have a system navigator [I-1] | | - |
|  |  |  | *Volunteers* | Recruiting enough volunteers [I-20] | | - Need to find enough skilled people to serve as volunteers [I-17] - In rural settings, volunteers could be relatives, friends, neighbors [I-15] - Build another category of volunteers, peer support volunteers, a person within their circle (between them or family or friend) that we train on the Health TAPESTRY approach [I-16]:   - *“I think moving out to other jurisdictions, maybe we build another category of volunteer where we peer support volunteers. It’s a person within their circle, among their family, it could be a friend, their son or daughter, that we train on the Health TAPESTRY approach, as far as the volunteer arm and the tool that we have offered the formal volunteer program and download that to sort of their peer support volunteer, that would be quite sustainable. I think because there wouldn’t be those barriers with traveling long-distances on bus or public transportation, they wouldn’t have to really look at personal safety, health and safety, confidentiality, things like that, so I think as we sort of move forward on this I guess that’s what’s happening here, it’s like a pet, it’s one of those boxes that you keep opening and there’s another box inside, with some other things to consider”* [I-16] |
|  |  |  |  | Expanding number of volunteers may change the team dynamic - they may not feel like they are part of a team [I-5] | |  |
|  |  |  |  | Not every physician office that will be involved in Health TAPESTRY will have the same resources or the capacity as the Hamilton site, particularly in terms of some of the operational things that are picked up by the volunteers [I-17] | |  |
|  |  |  |  | Getting the number of volunteers needed. If Health TAPESTRY opens up to more physicians, it will become very overwhelming very quickly [I-17, I-25] | |  |
|  |  |  |  | Recruiting students may be difficult in certain sites [as was in Hamilton] because of timing such as exams (at which time they are more busy) and not being around during the summer months [I-11, I-13] | | - Starting recruitment in the beginning of the University semester (September and October) to maximize availability of student volunteers [I-1] |
|  |  |  | *Volunteer coordination; volunteer organization* | A challenge for other sites might be scheduling and coordinating volunteer visits without a coordinator [I-11, I-13] | | - Having someone who has coordinated all the volunteers, and who has actually been able to lead the development of the volunteers and pilot work, who understands all the challenges will be very helpful with the roll-out elsewhere [I-1]   - *“For 10 clients it was manageable to coordinate volunteer schedules – but managing schedules for 300 clients and 50 or 60 volunteers would be a nightmare via paper”* [I-13] |
|  |  |  |  | Even though the training is going to be provided online, there will still be a need to have somebody to recruit them and direct them to the learning program [I-24] | |  |
|  |  |  |  | How to deal with different volunteer coordinators and how they communicate with family physicians [I-17] | |  |
|  |  |  |  | Volunteer organizations may not be available at other sites [I-1] | |  |
|  |  | Volunteers | | | | |
|  |  |  | *Engagement and retainment* | | Challenge will be retaining volunteers and keeping them engaged [I-11] | - |
|  |  |  | *Satisfaction* | | Increasing the size and number of volunteers might affect the sense of belonging as part of the Health TAPESTRY team and may also affect volunteers’ commitment to the program or they may not feel recognized or appreciated [I-5] | - |
|  |  |  | *Support* | | How to deal with volunteers that have questions [I-17] | - |
|  |  |  |  |  | It will be a challenge to provide ongoing support to volunteers in different locations across Canada [I-19] | - |
|  |  |  | *Training process* | | New volunteers may not get the same level of training as the pilot program [I5] | - Need to ensure that the quality of volunteer training is equivalent across the different trainers, how to deal with volunteers that have questions, and how to deal with different volunteer coordinators including how they communicate with family physicians [I-17] - In rural settings, it could work that somebody accesses the Health TAPESTRY training to understand what their roles, scope, and responsibilities are, someone that is authorized or approved by the primary care physician or nurse practitioner, and work together so that this relationship could be built [I-15] - Provide training that would be widely applicable to different types of client visits [I-5] - Need to train volunteers to deal with diversity or how to accommodate diversity [I-5] - Having volunteers who are multilingual or who can do some translation [I-5]: “*I think when we expand, I think it’s important that this program reaches diversities. For example, in my community, it’s a very Chinese based community, and I don’t think the program such as this as we see it right now would be able to work itself in there just because of the language barrier primarily…”* [I-5] |
|  |  |  |  |  | The volunteers trained at the Hamilton site are formalized – they are recruited, screened and interviewed. Moving on to other jurisdictions, this formalized process may not be possible [I-16] |  |
|  |  |  |  |  | How volunteers will be trained and supported in other settings may be a challenge [I-1, I-19] |  |
|  |  |  |  |  | During the training, the clients were homogeneous, but as Health TAPESTRY expands, there will be more diversity in the client encounters including the potential for different languages [I-5] |  |
|  |  |  |  |  | It will be a challenge to ensure that the quality of training is equivalent across the different sites [I-17] |  |
|  |  |  | *Client visits* | | In-person volunteer visits may take more time in other sites [I-3] | Make sure the number of clients per volunteer is reasonable (suggest 1-2/volunteer) so that the volunteers don’t have to go to so many visits per week, and so they don’t feel overworked and underappreciated – to keep the volunteer numbers reflective of the expanding client numbers [I-5] |
|  |  |  |  |  | If volunteers are asked to see too many clients [I-5] |  |
|  | **Site technology** | | | | | |
|  |  | Data adaptation | | Every site might have their own survey that they want to add on, which means that they will have to hire their own programmer to create custom reports [I-4] | | - Need to think about how the reports will be automated to create different reports depending on the needs of different sites [I-4] - Health TAPESTRY is server application, which can be used in many different situations, it doesn’t just have to be for Tapestry. The survey application are being designed to go to not just specifically for Tapestry [I-4] - Having an application that Tapestry creates that can be reused by different programs [I-4]: *“Having an application that Tapestry creates that can be reused by different programs, that’s a pretty good outcome I think”* [I-4]. |
|  |  | Data sharing | | Unclear what and how data will be shared with other sites [I-2] | | - |
|  |  | Hardware and software | | Size of the Tablet screens may be different in different sites, which may also affect the operating system, different restrictions, and the Health TAPESTRY application may not work as well on that browser [I-4] | | - Application will be native to the operating system so it can be accessed by any tablet or laptop [I-4] - There has to be a lot of computer synchronization or consistency across software systems for it to work everywhere [I-18] |
|  |  | Personal health record (PHR) | | It will be difficult to apply Health TAPESTRY in settings without a PHR, a different PHR or if the concept of a PHR is very new [I-6] | | - |
|  |  | Technology readiness | | Technology is a key component of Health TAPESTRY, so it will be limiting and complicated for paper-based primary care practices and those that don't have technology or limited technology, particularly in terms of how data will be gathered [I, 6, I-15, I-16] | | - |
| **3. Scale-up Process** | | | | | | |
| **Adaptability of Health TAPESTRY** | | | | | | |
|  | Consistency | | | How to keep the Health TAPESTRY program consistent throughout the different sites or provinces that will adopt them [I-5] | | - Make sure that Health TAPESTRY communicates the same things, but allow room for change but keep it consistent enough so that it is still Health TAPESTRY [I-15]:   - “*Making sure that the volunteers still do the same thing but allowing room for change but keeping it consistent enough from place to place so that it is still Health TAPESTRY, something that hasn’t morphed into something that strays from the original goals”* [I-5]   - *“Every city or province will have to modify Health TAPESTRY a little bit to suit their particular population, such as transportation“* [I-5] |
|  |  |  |  | Communicating the whole Health TAPESTRY message and values consistently across sites [I-11] | |  |
|  | Existing culture | | | Challenge of bringing new ideas and a new program into an already existing culture; how to create change culture and how does it get embedded in the culture of different organizations [I-1, I-3]; | | - It will be about the other site adapting an approach that they will make the determination of how that fits within their workflow [I-10] - Important to figure out how to integrate and build on work that is already being done and to be able to offer some of the core ideas and principals that makes Health TAPESTRY innovative [I-3] - Health TAPESTRY should be framed to other sites in a way that will let them know that the goal is not to replace or take over anything that is already happening there [I-3] - Health TAPESTRY will need to be tailored to the context of the setting; to support this tailoring, to other sites, need to identify the best practices or the principles of the process of doing things (e.g., finding an organization which has certain qualities that can be relied on or to identify the qualities that are needed to find to set up the volunteer system [I-12] - Identify key ingredients required to adapt Health TAPESTRY and the process for finding those key ingredients – this will also show the feasibility of what will be needed within a particular jurisdiction [I-12]: *“You’ll have to look at the assets in your neighborhood to see how you can make it work in your neighborhood. If you don’t have those same assets, maybe it’s not feasible”* [I-12] - Need to build something where Health TAPESTRY is led into other best practice initiatives or health care renewal initiatives that are being done [I-15] - Have a conversation with the clinic about the changes that will need to be put into place and how to deal with this change in the short and long term [I-15] |
|  | Feasibility | | | Adapting Health TAPESTRY beyond Canada would be difficult because of different health care systems [I-19] | | - Other countries are using volunteers and focusing on health technology, and there is a good recognition of the importance of the healthcare team and some kind of navigation role within the team [I-19] |
|  |  |  |  | It might take time to really know the other site, to learn and get information about how the system works there [I-6] | |  |
|  | Use of core Health TAPESTRY elements | | | The core components of Health TAPESTRY have not been established [I-2, I-3, I-6, I-10, I-14, I-15, I-19] | | - Need to know the core components of Health TAPESTRY and use pieces that are valuable and fit within their setting; in Canada, there should be a set of components that would be applicable across multiple sites and settings [I-10, I-19]:   - *“Think of Health TAPESTRY as yet another approach to or a number of components that could help optimal aging and for people to take those and marry them with things they are already doing will be useful – to create ways for people to look at what we have done [Health TAPESTRY] and then easily pull pieces to put them into their context”* [I-15] - The core components that would allow Health TAPESTRY to be scalable much more broadly are:   - Volunteers going out to the homes [I-19]   - The use of a PHR [I-19]   - The use of an IP team [I-19]   - Some kind of navigation through the system [I-19] - Adaptability of Health TAPESTRY in a way that its main objective or core concept is still there [I-6]: “The soul of Health TAPESTRY should be there, but definitely its body or frame can change depending on the resources that are there in the region already” [I-6] - Use the 4 pillars of Health TAPESTRY to make it more applicable to other sites and communities [I-14] - Determine what will be part of the Health TAPESTRY brand and how flexible each community could be in terms of making adaptations to their own [I-14] - Having a discussion with clinic leadership and those delivering the clinical care to share potential challenges and discuss how there might be other ways to adapt the process to maximize the value of information that is coming from the one element of Health TAPESTRY to the clinic [I-15] - Other sites may not adopt all the elements of what Health TAPESTRY may end up being, but they may see some really good components of it that can be adopted to their particular environment [I-2] |
|  |  |  |  | Clinicians may not adapt all of the elements of Health TAPESTRY; not all sites will going to be interested in all aspects of Health TAPESTRY because it’s so big [I-2, I-24] | |  |
| **Implementability of Health TAPESTRY** | | | | | | |
|  | Implementation guide/manual | | | Currently, no user manual on how to scale up; nothing to offer other sites [I-8, I-22] | | - There should be a package, implementation guide/manual/protocol to give to other sites that provides practical information (not just what to do but how to do it); it should be a web-based product that includes [I-2, I-4, I-8, I-15, I-22, I-25]:   - How to recruit volunteers   - How to train volunteers   - How to use online volunteer management process and virtual learning centre   - How to overcome risk management   - How to set up accounts for patients   - The type of hardware needed (laptops and tablets) they would need to use   - The technology requirements (hardware, servers)   - Who should be involved for each step and what are their responsibilities   - Do they need their own team to set up IT or do they want the Hamilton site to host it   - A description of what Tapestry offers (what it can do)   - A description of how to start and how to progress   - Structured so that other sites can share their own concepts and compare   - Include the number of decisions that were made and the rationale for those decisions to share with other sites   - A description of learnings from the Hamilton site Health TAPESTRY   - The guide should be available for free to any community in Canada |
|  |  |  |  | If the implementation plan is to rigid then Health TAPESTRY will be less scalable and more difficult to fit into other sites [I-10] | | - Need to present Health TAPESTRY as an approach with some pillars but recognize that it might be implemented in different ways - this will create more interest as the adopters can highlight the elements that could work for them [I-10] - It’s important to give sites some space to let things unfold in some ways and not push people because we want this to be something that they embrace and fit into what they are already doing [I-10] - It’s necessary to keep flexibility of what the core components might look like in different areas such as rural sites [I-19] |
|  | Implementation strategy | | | | | |
|  |  | Currently, no implementation strategy to offer other sites [I-8, I-22] | | | | - Develop some type of program implementation strategy which will help look at a small community versus a large community and give them tips, recommendations, how to do things, as well as to describe what Hamilton did, so now this is what you need to consider [I-16] |
|  |  |  | Customizability and fit | | | - Come up with an approach to scalability that will fit and is tailorable and adaptable to each locale in terms of their needs [I-10] - There will be a standard set of measures that each site will use, but because of the context of their own work and setting, they will measure things that are particular to them [I-15] - Need to understand how to work with patients not in the clinicians’ environment but in the patient’s environment, which is a new way of thinking about it [I-2] - Sites should use the pieces of Health TAPESTRY that are valuable and fit within their setting; to use an approach whereby a number of Health TAPESTRY components related to optimally aging and marry them with things that the sites are already doing, something that will be useful; to create ways for people to look at what Health TAPESTRY has done and then pull pieces and put them into their current context [I-10, I-15]   - *“So you know, not all areas are going to be interested in all aspects of Tapestry because it’s so huge, but I think that that’s the beauty id that people will be able to take the parts that they need or that they feel that they can work with and apply it and learn from what’s happening here in terms of what might be useful for them.” [I-24]* |
|  |  |  | Key learnings from Hamilton site | | | - People will be able to take the parts that they need or that the feel that they can work with and apply it and learn from what’s happening in the Hamilton site and apply what might be useful for them [I-24] - Understand what has been some of the key drivers of what has worked so far [I-15] - Figure out how to transfer information about what has worked so far and make available to other groups, and learn from other groups who are about to start implementing Health TAPESTRY [I-15]   - *“I also think that the other skill set that the Tapestry team has is this ability to say no we don’t have all the answers, we are working through this process, we are not exactly sure all the things that we are going to encounter along the way, but if you encounter something and learn something, please teach us and we will be happy to share what we are learning as well, so it has some real inherent flexibility and I think that that is the spirit you need to spearhead great ideas”* [I-24]. |
|  |  |  | Monitoring | | | - To be adopted, Health TAPESTRY needs to be in place so that it can be monitored, to make sure it’s being delivered in the right way and that it’s meeting certain targets [I-2] |
|  |  |  | Needs assessment | | | - It would be important to conduct a needs assessment across the country to see how the Health TAPESTRY model may fit elsewhere, to better understand what the potential for uptake, to better understand their health system and environment, what are their existing models of care, because provinces can vary quite substantially in this regard [I-1] |
|  |  |  | Onsite champion | | | - There could be a champion identified onsite that could be motivated to take things on [I-23] - There needs to be a lead or a clinician in a remote area that wants to roll out Health TAPESTRY [I-8] |
|  |  |  | On-site mentoring and training | | | - Provide onsite mentoring and training of physicians with their own patients such as using peer-to-peer education or peer-to-peer advantages will help increase capacity and scale [I-23] |
|  | Relationship with adopter site | | | | | |
|  |  | How to nurture relationships with other sites doesn’t exist [I-3] | | | | - Need to find ways to nurture and grow relationships with adopter sites [I-3] - To have someone (similar to the Hamilton site) that can trouble shoot and coordinate at other sites just initially until things are set up [I-1] - It’s important to have outreach and its coordination from the Hamilton team in helping to implement it elsewhere [I-22] |
|  |  | Geography is going to be a challenge and take time – the fact that Health TAPESTRY team won’t have close contact and same level of interaction [I-3] | | | |  |
